# C10orf55, CASC2, and SFTA1P lncRNAs Are Potential Biomarkers to Assess Radiation Therapy Response in Head and Neck Cancers

**DOI:** 10.3390/jpm12101696

**Published:** 2022-10-11

**Authors:** Anna Paszkowska, Tomasz Kolenda, Kacper Guglas, Joanna Kozłowska-Masłoń, Marta Podralska, Anna Teresiak, Renata Bliźniak, Agnieszka Dzikiewicz-Krawczyk, Katarzyna Lamperska

**Affiliations:** 1 Laboratory of Cancer Genetics, Greater Poland Cancer Center, Garbary Street 15, 61-866 Poznan, Poland; 2 Faculty of Biology, Adam Mickiewicz University, Umultowska 89, 61-614 Poznan, Poland; 3 Research and Implementation Unit, Greater Poland Cancer Center, Garbary Street 15, 61-866 Poznan, Poland; 4 Postgraduate School of Molecular Medicine, Medical University of Warsaw, Zwirki and Wigury Street 61, 02-091 Warsaw, Poland; 5 Institute of Human Biology and Evolution, Faculty of Biology, Adam Mickiewicz University, Uniwersytetu Poznańskiego 6, 61-614 Poznan, Poland; 6 Institute of Human Genetics, Polish Academy of Sciences, Strzeszynska 32, 60-479 Poznan, Poland

**Keywords:** C10orf55, CASC2, SFTA1P, lncRNA, ncRNA, HNSCC, biomarker, radiotherapy, TCGA

## Abstract

Long non-coding RNAs have proven to be important molecules in carcinogenesis. Due to little knowledge about them, the molecular mechanisms of tumorigenesis are still being explored. The aim of this work was to study the effect of ionizing radiation on the expression of lncRNAs in head and neck squamous cell carcinoma (HNSCC) in patients responding and non-responding to radiotherapy. The experimental model was created using a group of patients with response (RG, *n* = 75) and no response (NRG, *n* = 75) to radiotherapy based on the cancer genome atlas (TCGA) data. Using the in silico model, statistically significant lncRNAs were defined and further validated on six HNSCC cell lines irradiated at three different doses. Based on the TCGA model, C10orf55, C3orf35, C5orf38, CASC2, MEG3, MYCNOS, SFTA1P, SNHG3, and TMEM105, with the altered expression between the RG and NRG were observed. Analysis of pathways and immune profile indicated that these lncRNAs were associated with changes in processes, such as epithelial-to-mesenchymal transition, regulation of spindle division, and the p53 pathway, and differences in immune cells score and lymphocyte infiltration signature score. However, only C10orf55, CASC2, and SFTA1P presented statistically altered expression after irradiation in the in vitro model. In conclusion, the expression of lncRNAs is affected by ionization radiation in HNSCC, and these lncRNAs are associated with pathways, which are important for radiation response and immune response. Potentially presented lncRNAs could be used as biomarkers for personalized radiotherapy in the future. However, these results need to be verified based on an in vitro experimental model to show a direct net of interactions.

## 1. Introduction

Head and neck squamous cell carcinomas (HNSCC) are the sixth most common among all cancers worldwide. The mortality rate of HNSCC patients can be as high as 50% [1]. On this basis, two groups were distinguished among HNSCC: cancers caused by carcinogens, such as alcohol and smoking, which account for up to 75% of the incidence of HNSCC, and those associated with a viral infection, such as HPV [2,3,4,5]. A patient’s treatment is determined after evaluating factors, such as the size of the tumor, the presence and number of metastases, and their location. Despite medical advances, the mortality rate among HNSCC patients is still high [6]. Due to the low clinically apparent precancerous changes, in most cases, cancer is diagnosed at a late stage, which reduces the chances of recovery [7]. Current strategies for treating HNSCC include surgical and non-surgical approaches, such as radiation therapy. For patients with head and neck cancers, conventional radiation therapy is used, during which patients are subjected to radiation at a fraction of 2 Gy once a day until the total radiation dose received is 70 Gy [8]. Molecular mechanisms of HNSCC pathogenesis, however, have still not been fully clarified, so it is crucial to investigate their genetic basis and to improve diagnostic methods and treatment [9].

Among HNSCC patients, radiation therapy is a widely used treatment method. As studies have shown; however, it has proven to be more effective in HPV-positive patients than in HPV-negative patients [10,11,12]. The susceptibility of this group of patients is due to abnormalities in signaling and repair mechanisms within DNA strands. On this basis, much of the current research focuses on the protein-coding genes and proteins themselves, which are responsible for repair mechanisms and inhibitors associated with cell cycle regulation. One of the consequences of ionizing radiation, with a profound impact on cell function, is DNA damage, and consequently a whole cascade of repair systems. Following irradiation, signaling pathways are activated to repair the damage or apoptotic mechanisms are triggered [13]. DNA damage can affect one or both DNA strands, and thus single-strand breaks (SSBs) and double-strand breaks (DSBs) are distinguished. DSBs are characterized by impaired DNA repair kinetics and numerous oxidative base impairment, resulting in genome destabilization. Additionally, an important effect of radiation on the cell is water ionization, which causes the formation of free radicals in the cell, which are harmful to the genetic material of the cell and can lead to cell death [14]. However, it is possible that after a dose of infrared radiation, DNA repair cascade effectively and largely restores the functionality of cancer cells, and as a result, these cells can acquire radioresistance [15,16]. It was shown that under the influence of ionizing radiation and chemotherapeutics expression not only of protein-coding genes but also of non-coding RNAs is changed as a natural response and leading to molecular changes, which can overcome harmful factors [17].

Although long non-coding RNAs (lncRNAs) do not encode proteins, they have been recognized as valuable and significant molecules over time. Their sequence consists of more than 200 nucleotides and very often contains elements typical of mRNA, namely poly-A tails and regulatory elements, such as miRNAs binding sites. The process of lncRNA biogenesis is regulated by RNA polymerase II and is similar to mRNA formation [18]. The activity of lncRNAs is characterized not only by interaction with proteins and other RNAs but also by regulation of transcription and expression of genes through changes in chromatin structure [19,20].

lncRNAs appear to be important in functions related to the regulation of gene transcription in the nucleus or subsequent post-transcriptional modifications in the cytoplasm [21]. Abnormalities in the activity or biogenesis mechanisms of lncRNAs can appear in states of pathological conditions and indicate cancer progression by affecting not only the structure of the chromatin but also several transcription factors [22]. It has been demonstrated that lncRNAs play a key role in cancer biology. Although the function and activity of lncRNAs are still under investigation, in the future they may become important tools for predicting the development and possible treatment of cancer [23]. Moreover, dysregulated lncRNAs are closely associated with the regulation of a cellular pathway associated with the response to irradiation [16]. A better understanding of the mechanisms of tumor cell response to radiation therapy, and characterization of the genes, including lncRNAs, which can be used as irradiation markers, gives the possibility of more effective treatment.

**Figure 1:**
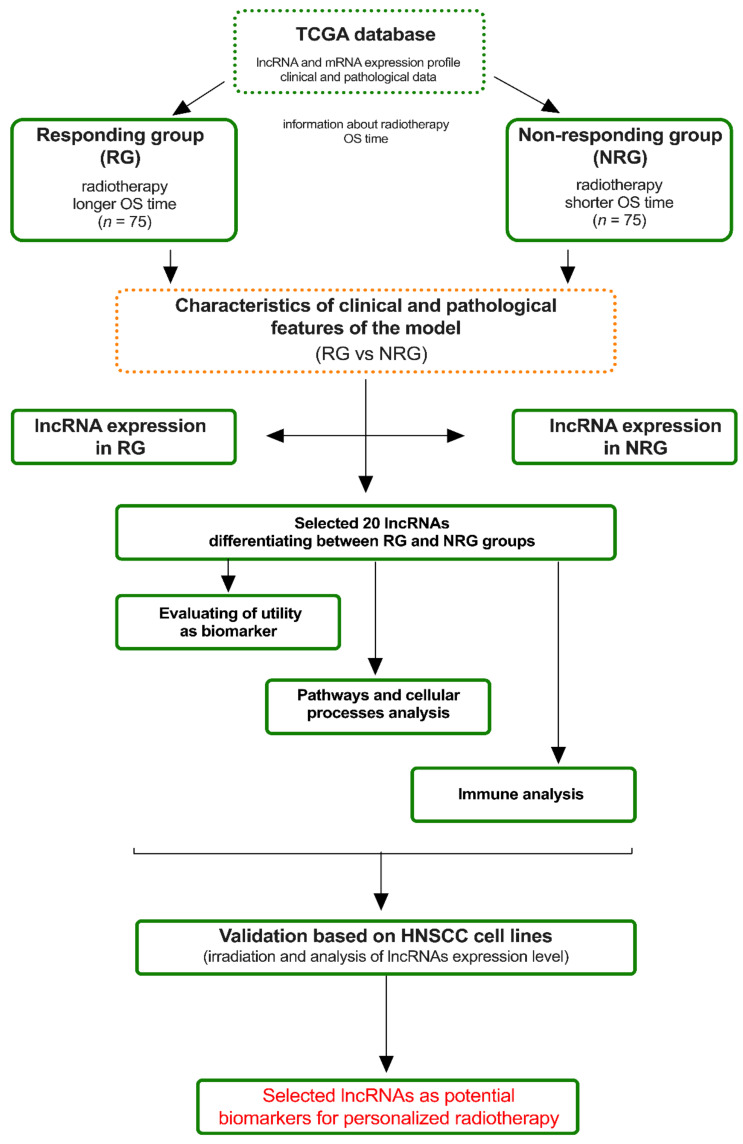
Overview of main experimental steps divided into in silico (based on The Cancer Genome Atlas (TCGA) analysis) and in vivo (cell lines analysis) used in this study.

In this study, we used The Cancer Genome Atlas (TCGA) data of HNSCC patients to define the lncRNAs’ panel with a high ability to distinguish patients in response to implemented radiotherapy during the treatment. Based on this, nine selected lncRNAs were validated using HNSCC cell lines and different doses of irradiation. The overview of the experimental approach with analysis steps used in this study is presented in Figure 1.

## 2. Materials and Methods

### 2.1. Databases

Publicly available data from the TCGA was downloaded from the website of the University of Santa Cruz in California (https://xenabrowser.net/datapages/, accessed on 12 March 2022). Files included gene expression and clinical presentation of patients with head and neck cancers: TCGA.HNSC.sampleMap/HiSeqV2 was used (with expression unit: estimated gene level transcription, log2(x + 1) transformed RSEM normalized count, number of patients: 566). lncRNAs of interest were selected using the tool BioMart available at: https://www.ensembl.org/index.html, European Bioinformatics Institute (EMBL-EBI), Wellcome Genome Campus, Hinxton, Cambridgeshire, United Kingdom, accessed on 12 March 2022.

ESTIMATE (Estimation of STromal and Immune cells in MAlignant Tumor tissues using Expression data) data for immune cell analysis were downloaded from https://bioinformatics.mdanderson.org/estimate/disease.html (University of Texas MD Anderson Cancer Center Houston, Texas, United States of America, accessed on 25 August 2022), platform RNA-Seq-V2 [24]. Data presented by Thorsson et al. [25] (accessed on 25 August 2022) was used for the analysis of lymphocyte infiltration signature score.

All the data are available online with unrestricted access and do not require the patients’ consent or other permissions. The use of the data does not violate the right of any person or any institution.

### 2.2. Obtaining the Model and Its Characterization

To build the model, 297 patients with radiation treatment were selected to obtain two groups of patients who were separated based on their overall survival (OS). These two marginal groups of patients were named further as the responding group with OS longer than 1049 days (RG) and the non-responding group with OS shorter than 854 days (NRG). The rest of the 147 patients with OS time less than 1049 days and higher with 854 were excluded (percentile 25–50% and 50–75%). Information about patients included in RG and NRG groups is enclosed in Supplementary Table S1. The obtained model of 150 patients (RG with *n* = 75 and NRG with *n* = 75) was characterized based on the clinical and pathological information in terms of criteria, such as location: oral cavity vs. pharynx vs. larynx; tumor cell differentiation grade: G1 vs. G2 vs. G3; tumor size: T1 vs. T2 vs. T3 vs. T4; HPV p16 status: positive vs. negative; alcohol consumption history: yes vs. no; smoking tobacco products history: yes vs. no, as described in the statistical section.

### 2.3. Pathways and Cellular Processes Analysis

Gene set enrichment analysis (GSEA) of a group of genes was conducted using software from www.gsea-msigdb.org, accessed on 10 May 2022. The tested 150 patients were divided into two groups with low and high expression of a particular lncRNA with the median expression used as a cutoff. The analysis took into account the expression profile of all genes for a given patient. Analysis of oncogenic signatures (C) from MSigDB collection with 1000 gene set permutations and with nominal *p*-value ≤ 0.05 and *FDR* q-value ≤ 0.27 were considered significant.

Next, the enriched gene sets for all significant results from GSEA were analyzed using the GeneMANIA online tool (https://genemania.org, accessed on 20 August 2022) for deeper prediction of their biological network integration for gene prioritization and function and visualization of possible interactions [26].

### 2.4. Immune Analysis

Infiltration of immune cells into tumor tissues and inference of the tumor purity in patients’ groups depending on the high and low lncRNA expression level (cut off based on the median expression of a specific gene) was analyzed using the dataset ESTIMATE [24]. Next, lymphocyte infiltration signature scores were estimated depending on the high and low expression levels of specified lncRNA in the whole (RG and NRG) group of patients using deconvolution data about specific immune cells presented by Thorsson et al. [25] as described in the statistical method section.

### 2.5. Irradiation of HNSCC Cell Lines

Commercially available head and neck squamous cell lines DOK, SCC-25, SCC-040, FaDu, CAL-27, and Detroit 562 were used for the analysis of changes in the expression level of specified lncRNAs selected based on the TCGA analysis. All cell lines were cultured in DMEM (BioWest, Nuaille, France) supplemented with 10% FBS (BioWest) and 4.5 g/L gentamicin antibiotic (Krka, Poland) in culture bottles and incubated in a 37 °C and 5% CO_2_ atmosphere.

The culture bottles were filled with PBS and the cells were exposed to ionizing radiation at doses of 2, 4, and 8 Gy, similar to those described previously by Lindell Jonsson E et al. [27], using Gammacell^®^ 3000 Elite (Theratronics, Canada) with the Cesium-137 isotope as the radiation source with the emission of α, β+ and β−, and gamma-penetrating radiation. As controls, cell lines that were identically grown but not subjected to ionizing radiation (0 Gy) were used. All irradiation experiments were made with a minimum of three independent biological replicates. After irradiation, the PBS was removed, and cells were cultured for 24 h as described above. After that, total RNA was isolated using TRI Reagent^®^ (Sigma-Aldrich, Munich, Germany) according to the manufacturer’s protocol. The quality and quantity of RNA were estimated by observation of 28S and 18S rRNA bands using electrophoresis in 1% agarose with TEA (Tris-acetate-EDTA (Ethylenediaminetetraacetic acid)) buffer and NanoDrop 2000 spectrophotometer (Thermo Scientific, Waltham, MA, USA).

### 2.6. lncRNA Expression Analysis

Reverse transcription was performed using EvoScript cDNA synthesis reaction kit (Roche, Basel, Switzerland) according to the manufacturer’s protocol and using 1 µg of total RNA. Obtained cDNA was diluted (10×) and used for a quantitative reverse transcription-polymerase chain reaction (qRT-PCR) using LightCycler 96 (Roche) and SYBR Green I Master buffer (Roche) as described previously [28].

The primers for quantification of C10orf55, C3orf35, C5orf38, CASC2, MEG3, MYCNOS, SFTA1P, SNHG3, and TMEM105 were described previously [28,29,30,31,32,33,34] and are presented in Table 1. The compatibility of all primers and their complementarity to the target sequence was checked using the Basic Local Alignment Search Tool (BLAST), National Center for Biotechnology Information (NCBI), Bethesda, Maryland, United States of America, available at: https://blast.ncbi.nlm.nih.gov/Blast.cgi, accessed on 15 May 2022. All real-time PCR data were analyzed by calculating the 2^−∆Ct^ method and normalized against the GAPDH expression as described previously [29,30,31,32,33,34,35].

### 2.7. Statistical Methods

All statistical analyses of the data extracted from TCGA-based databases were performed using the GraphPad Prism 5 software. For each of the analyzed groups, the distribution was checked using the Shapiro–Wilk test. Analysis of differences between groups was performed using the Student’s *t*-test or Mann–Whitney U test, depending on the distribution. Differences in clinical and pathological parameters in the groups of patients responding and non-responding to ionizing radiation were analyzed using the Chi-square test. Differences in the expression levels for individual lncRNAs in cell lines undergoing irradiation were analyzed using the ANOVA test. Previously, it was checked whether the normal distribution was present in each group. For the primary analysis, the Kruskal-Wallis ANOVA test was used, and for the post hoc analysis, the Dunn’s test. For all analyses, the test probability value (*p*-value) was assumed to be statistically significant, when it is less than 0.05.

For each analysis, it was assumed that the value of the test probability (*p*-value) is statistically significant when it is less than 0.05.

## 3. Results

### 3.1. Obtaining an In Silico Experimental Model Based on TCGA Data

**Figure 2:**
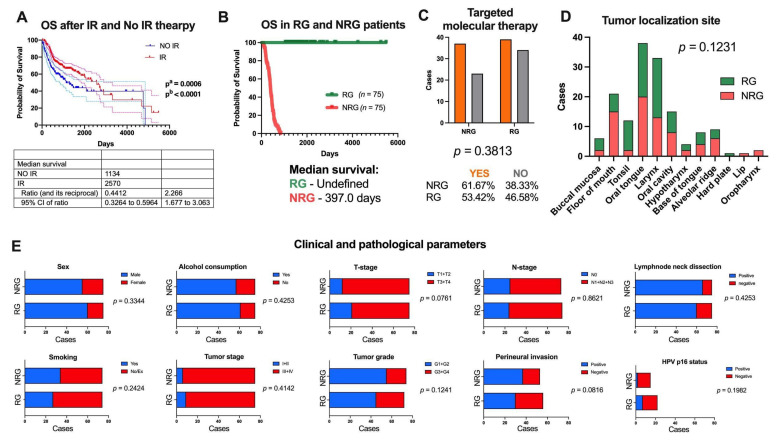
Characteristics of an in silico model obtained based on the TCGA head and neck squamous cell carcinomas (HNSCC) patients: (**A**) overall survival (OS) time of HNSCC patients depending on radiotherapy (IR, dark red) and no radiotherapy (No IR, dark blue) treatment with 95% CI marked as lighter lines; (**B**) OS time in responders group (RG, green) and non-responders group (NRG, red) of patients to radiotherapy; (**C**) percent of the patients who underwent targeted molecular therapy; (**D**) localization of tumors depending on the RG and NRG groups; and (**E**) main clinical and pathological parameters describing RG and NRG groups; *p* < 0.05 considered as statistically significant.

First, using the data available from the TCGA project the overall survival (OS) time of HNSCC patients with radiation therapy and without this treatment was assessed. It was observed that patients who received radiation therapy displayed prolonged OS time with a median survival of 2570 days in comparison to the group without this treatment with a median survival assessed at 1134 days (*p* = 0.0006 and *p* < 0.0001) (Figure 2A).

Next, from 297 patients who received radiation therapy, two distinct groups named responders’ (RG) and non-responders’ groups (NRG) were separated based on OS. The responding group was the group of patients with the longest survival time (5480 days, with undefined median survival) after the procedure was performed and consisted of 75 patients. The non-responders’ group was the group of patients with the shortest survival time (69 days with median survival assessed of 379 days) after the procedure was performed and included 75 patients (Figure 2B). None of the 150 patients had a history of neoadjuvant treatment. Moreover, information about additional pharmaceutical therapy (unknown type) was presented only for 9 patients from RG (3 patients received therapy and 6 patients without therapy) and 31 from NRG groups (14 patients received therapy and 17 patients without therapy). Based on Chi-square analysis, no differences between groups were observed (*p* = 0.5274). The lack of information about additional pharmaceutical therapy is for 94% of RG and 79% of NRG patients in the TCGA data. Moreover, no differences in the number of patients undergoing targeted molecular therapy among RG and NRG patients were observed (*p* = 0.3813); see Figure 2C. Additionally, no differences in tumor localization sites in the RG and NRG group of patients were noticed (*p* = 0.1231); see Figure 2D.

The differences between the responding and non-responding groups to radiotherapy were examined based on selected clinical and pathological parameters. It was observed that there were no differences in several parameters: gender (female vs. male, *p* = 0.3344), alcohol consumption (yes vs. no, *p* = 0.4253), smoking of tobacco products (yes vs. no, *p* = 0.2424), stage of tumor (I + II vs. III + IV, *p* = 0.4142), tumor cell differentiation stage (G1 + G2 vs. G3 + G4, *p* = 0.1241), tumor size (T1 + T2 vs. T3 + T4, *p* = 0.0761), the presence of tumor cells in lymph nodes (N0 vs. N1 + N2 + N3, *p* = 0.8621), invasion of the perineural space (yes vs. no, *p* = 0.0816), degree of spread of tumor cells (I + II vs. III + IV, *p* = 0.1241), excision of cervical lymph nodes (yes vs. no, *p* = 0.4253), and HPV infection status (positive vs. negative, *p* = 0.1982). All results are shown in Figure 2E and Supplementary Table S2.

### 3.2. lncRNA Expression Profile Differs between RG and NRG Patients

**Figure 3:**
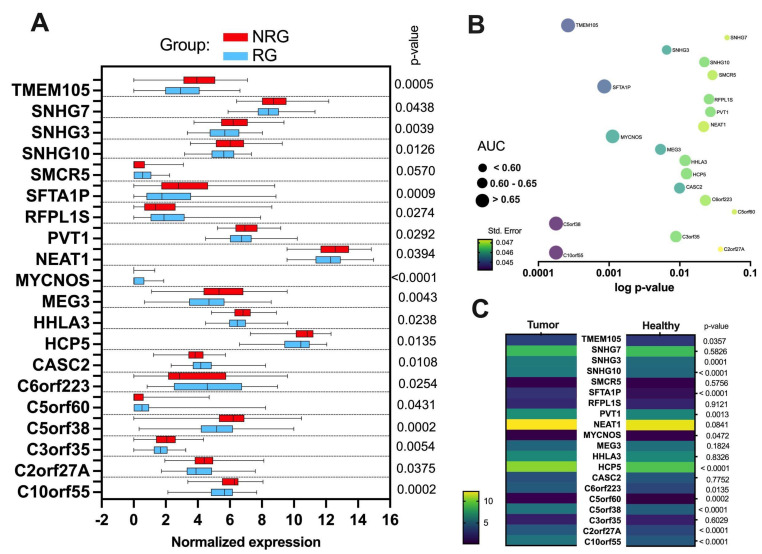
lncRNA expression profile (**A**) in responders’ group (RG) and non-responders’ group (NRG) of patients to radiotherapy and (**B**) assessment of the parameter AUC (Area under the ROC Curve) to discriminate the RG and NRG groups. (**C**) Comparison of expression levels of twenty lncRNAs between tumor (*n* = 522) and healthy (*n* = 44) using all HNSCC data taken from the TCGA; Student *t*-test or Mann-Whitney U test, *p* < 0.05 considered statistically significant.

Significantly different (*p* < 0.05) expression between RG and NRG patients was observed for twenty lncRNA. Next, C10orf55, C3orf35, C5orf38, CASC2, MEG3, MYCNOS, SFTA1P, SNHG3, and TMEM105 were selected for further analysis as the most changed between the group of RG and NRG patients. Increased expression in the NRG was observed for C10orf55 (*p* = 0.0002), C3orf35 (*p* = 0.0054), C5orf38 (*p* = 0.0002), TMEM105 (*p* = 0.0005), MEG3 (*p* = 0.0043), SFTA1P (*p* = 0.001), and SNHG3 (*p* = 0.0039), and lower expression in the case of MYCNOS (*p* = 0.0001), as well as CASC2 (*p* = 0.01). (Figure 3A and Supplementary Table S3).

Analysis of the receiver-operating characteristic (ROC) curve was performed to describe lncRNAs’ ability to differentiate groups of patients (RG vs. NRG). It was observed that 17 lncRNAs displayed AUC higher than 0.6 and in this group MYCNOS (AUC = 0.6544; *p* = 0.0013), SFTA1P (AUC = 0.6577; *p* = 0.001), TMEM105 (AUC = 0.672; *p* = 0.0003), C5orf38 (AUC = 0.6777; *p* = 0.0002), and C10orf55 (AUC = 0.6784; *p* = 0.0002) had the higher ability to differentiate patients with the response to radiotherapy (Figure 3B and Supplementary Table S4).

Moreover, the expression levels of these twenty lncRNAs were compared between tumor (*n* = 522) and healthy samples (*n* = 44) using all HNSCC data taken from the TCGA. It was indicated that expression levels were up-regulated for SNHG3, C10orf55, C2orf27A, C5orf38, HCP5, SFTA1P, SNHG10 (for all *p* < 0.0001), C5orf60 (*p* = 0.0002), PVT1 (*p* = 0.0013), C6orf223 (*p* = 0.0135), and TMEM105 (*p* = 0.0357) and downregulated for MYCNOS (*p* = 0.0472) in tumor compared to healthy samples. No differences were observed for NEAT1 (*p* = 0.0841), MEG3 (*p* = 0.1824), SMCR5 (*p* = 0.5756), SNHG7 (*p* = 0.5826), C3orf35 (*p* = 0.6029), CASC2 (*p* = 0.7752), HHLA3 (*p* = 0.8326), and RFPL1S (*p* = 0.9121); see Figure 3C and Supplementary Table S5.

### 3.3. Selected lncRNAs Are Connected with Pathways and Cellular Processes Important for Response to Irradiation

Next, pathways and cellular processes analysis for the nine lncRNAs showing the greatest differences in the RG and NRG groups was carried out. Gene set enrichment analysis (GSEA) was performed depending on the high and low expression levels of specified lncRNA using the set of 150 patients (RG and NRG groups).

**Figure 4:**
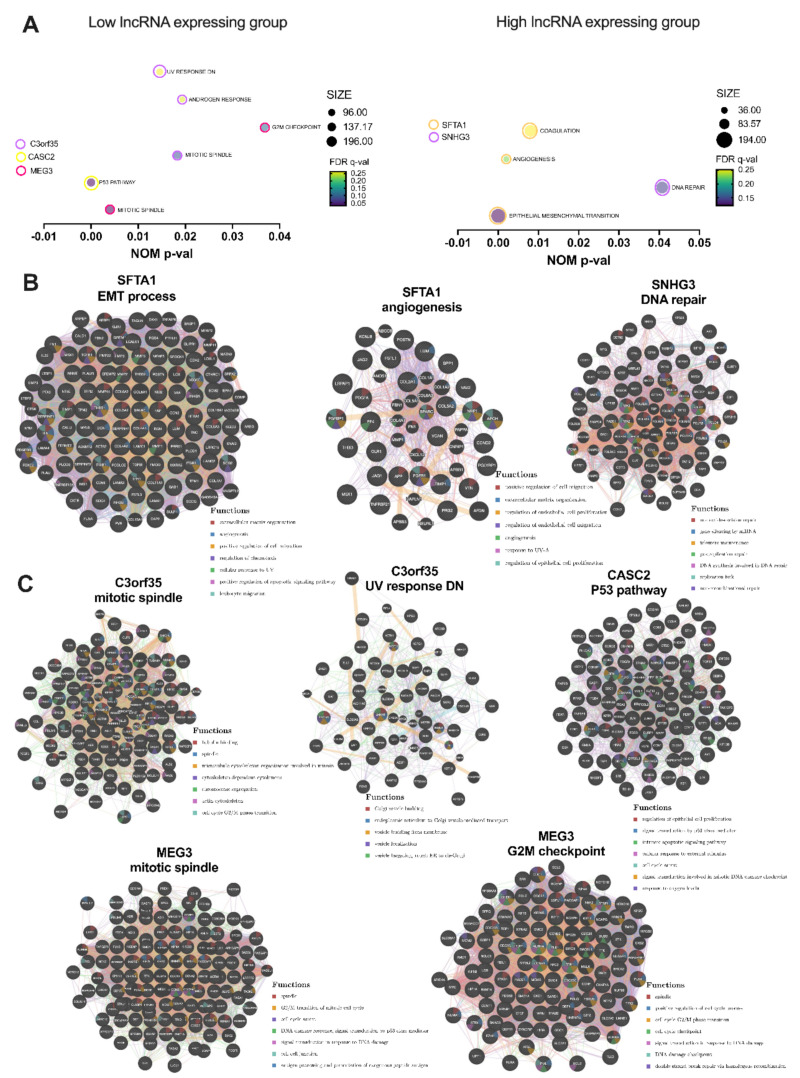
Pathways and cellular processes associated with expression level of C3orf35, CASC2, MEG3, SFTA1P, and SNHG3 lncRNAs: (**A**) Gene set enrichment analysis (GSEA) analysis of genes enriched in the set of 150 HNSCC patients (RG and NRG groups) depending on expression level of specified lncRNA (high or low groups divided based on the median expression of lncRNA); only the gene sets with nominal *p* < 0.05 were presented; *NES*-normalized enrichment score, *FDR* q-value-false discovery rate, SIZE-number of enriched genes in a specified process; and further analysis of genes included in GSEA results (*FDR* < 0.27 and *p* < 0.05) for (**B**) genes enriched in the group of patients with higher level of SFTA1 and SNHG3 lncRNAs, and (**C**) lower level of C3orf35, CASC2, and MEG3, and connected with ionization radiation response; GeneMANIA tool; *p* < 0.05 considered as statistically significant.

Based on the GSEA analysis altered peroxisome-associated pathways were observed for lncRNA CASC2 associated with the P53 pathway (190 genes, *FDR* = 0.030, *p* < 0.0001); for C3orf35 associated with response to androgen hormones (96 genes, *FDR* = 0.254, *p* = 0.019), UV response (137 genes, *FDR* = 0.264, *p* = 0.016), and the mitotic spindle (196 genes, *FDR* = 0.088, *p* = 0.018); for SFTA1P associated with coagulation (136 genes, *FDR* = 0.251, *p* = 0.008), epithelial-to-mesenchymal transition (194 genes, *FDR* = 0.122, *p* < 0.0001), and angiogenesis (36 genes, *FDR* = 0.226, *p* = 0.002); for MEG3 associated with the G2M checkpoint (184 genes, *FDR* = 0.108, *p* = 0.0367), mitotic spindle (196 genes, *FDR* = 0.030, *p* = 0.004); for SNHG3 associated with DNA repair (139 genes, *FDR* = 0.144, *p* = 0.041). All results are shown in Figure 4A and Supplementary Table S6.

Next, using the GeneMANIA tool, further analysis of genes included in GSEA results (*FDR* < 0.27 and *p* < 0.05) was performed and included the following processes: epithelial-to-mesenchymal transition process and angiogenesis for SFTA1 and DNA repair for SNHG3 observed in the group of patients with higher levels of these lncRNAs. The same analysis was conducted for enriched processes in the patients with lower expression of C3orf35 (mitotic spindle, UV response DN), CASC2 (TP53 pathway), and MEG3 (mitotic spindle and G2M checkpoint). For SFTA1, additional detected processes included: extracellular matrix organization, angiogenesis, cellular migration, response to UV, apoptosis, and connection with leukocyte migration, extracellular matrix organization, angiogenesis, response to UV, or regulation of cell proliferation. In the case of SNHG3 were noticed processes associated with DNA repair (e.g., nucleotide-excision repair, telomere maintenance, DNA synthesis involved in DNA repair) as well as gene silencing by miRNA. Analysis for lower expression of C3orf35 indicated changes in processes associated with Golgi apparatus and vesicle transport. Moreover, with this lncRNA processes strictly connected with division, cytoskeleton, and cell cycle were observed. These processes were also noticed with MEG3 and additional processes associated with DNA damage response and signal transduction caused by changes in DNA, cell–cell junction, as well as processes linked with antigen processing and presentation were also noticed. Patients with lower levels of MEG3 displayed also changes in processes, such as a mitotic spindle, cell cycle regulation, signal transduction in response to DNA damage, DNA damage checkpoint, and double-strand break repair via homologous recombination. The last lncRNA, CASC2, was connected with the regulation of proliferation, signal transduction, apoptosis, cell cycle arrest, response to external stimulus, signal transduction involved in mitotic DNA damage checkpoint, as well as response to oxygen levels. All results are presented in Figure 4B,C, and Supplementary Table S7.

**Figure 5:**
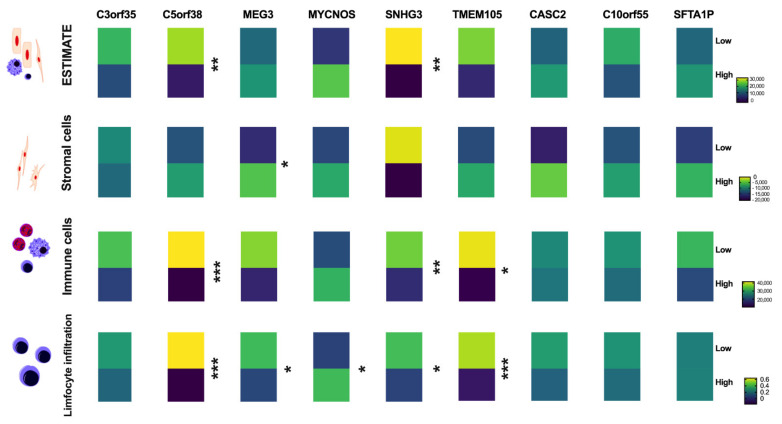
Analysis of infiltration of immune cells into tumor tissues and inference of the tumor purity depending on the expression level of CASC2, C10orf55, and SFTA1P; Student *t*-test or Mann-Whitney U test, *p* < 0.05 considered significant, ns-no significant; * *p* < 0.05; ** *p* < 0.01; *** *p* < 0.001.

Infiltration of immune cells into tumor tissues and infer tumor purity depending on the lncRNA expression level was analyzed using the dataset ESTIMATE because immune processes were observed for some of the pathways associated with selected lncRNAs. No changes between high and low expression levels of C3orf35, MEG3, MYCNOS, TMEM105, CASC2, C10orf55, and SFTA1P (*p* > 0.05) were observed. However, for C5orf38 and SNHG3, differences in tumor purity between patients with low and high levels of these two lncRNAs were indicated (*p* = 0.0005 and *p* = 0.0013, respectively). Only in the case of MEG3 differences in stromal cells were noticed (*p* = 0.0122). Lower levels of immune score and lower levels of lymphocyte infiltration signature score in the group of patients with higher levels of C5orf38 (*p* < 0.0001 and *p* < 0.0001, respectively), SNHG3 (*p* = 0.0093 and *p* = 0.0243, respectively) as well as TMEM105 (*p* = 0.0268 and *p* = 0.0002, respectively) in comparison to patients with lower levels of these lncRNAs were observed. Moreover, in the case of patients with lower levels of MEG3 and higher levels of MYCNOS, a higher lymphocyte infiltration signature score was observed (*p* = 0.0334 and *p* = 0.0475, respectively). All results are shown in Figure 5 and Supplementary Table S8.

### 3.4. Expression Level of lncRNAs Selected Based on TCGA Data WasChanged after Ionization Radiation in Cell Line Models

Finally, the C10orf55, C3orf35, C5orf38, CASC2, MEG3, MYCNOS, SFTA1P, SNHG3, and TMEM105 lncRNAs, were validated using HNSCC cell line models, which were irradiated using 2, 4, and 8 Gy of doses and compared to the non-irradiated controls (0 Gy). Changes in the expression were assessed using the 2^−∆Ct^ method and normalized to the GAPDH reference gene [28,29,30,31,32,33,34].

**Figure 6:**
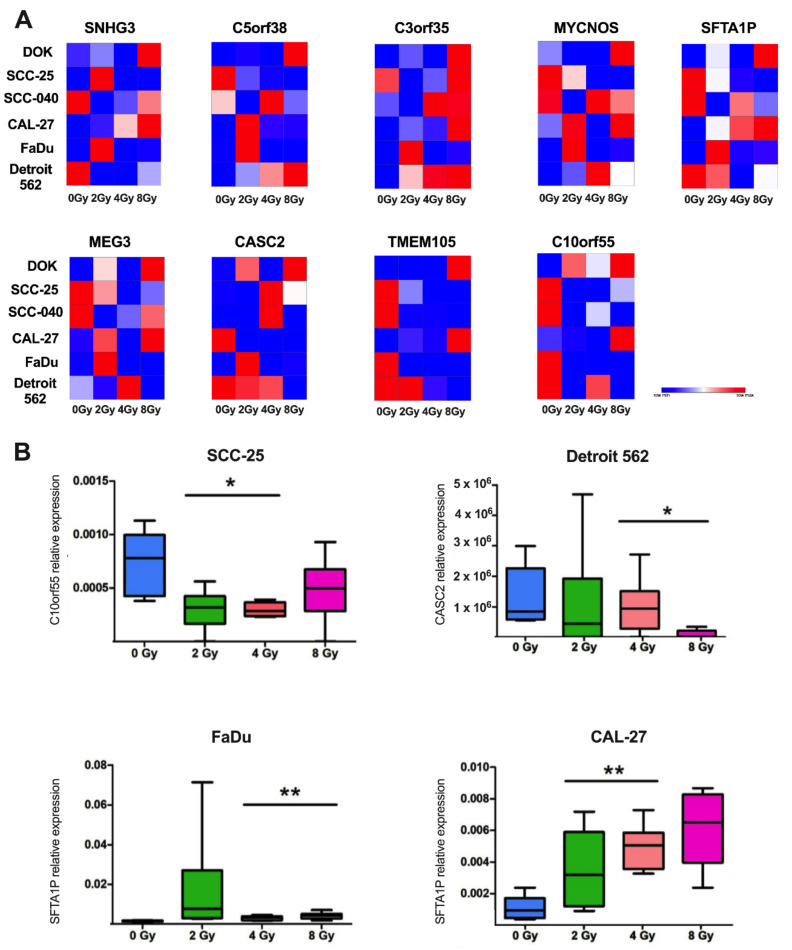
Relative expression of C10orf55, C3orf35, C5orf38, CASC2, MEG3, MYCNOS, SFTA1P, SNHG3, and TMEM105 lncRNAs after irradiation of HNSCC cell lines using doses of 0 Gy, 2 Gy, 4 Gy, and 8 Gy: (**A**) heat maps representing expression of specified lncRNAs and (**B**) expression of C10orf55, CASC2, and SFTA1P in selected cell lines; *p* < 0.05 considered significant, * *p* < 0.05; ** *p* < 0.01.

We observed that the expression level of C10orf55 was downregulated in SCC-25 cells after irradiation (*p* = 0.0146). In the case of CASC2 lncRNA in Detroit 562 cells downregulation of this lncRNA was observed (*p* = 0.0482). Opposite results were determined for SFTA1P, whose expression levels were up-regulated in CAL-27 (*p =* 0.0057) and FaDu cells (*p =* 0.0043) after irradiation. For the rest of the cell lines and specified lncRNAs, no significant differences were noticed (*p* > 0.05). All results are presented in Figure 6.

## 4. Discussion

Because the survival rate of the HNSCC patient group is still poor, understanding the genetic basis is crucial to finding personalized treatment and potential biomarkers for HNSCC. The present work focuses on investigating the relevance of specific lncRNAs in the pathomechanisms of head and neck cancers after ionizing radiation. In the context of tumorigenesis, the literature data on the role of lncRNAs in head and neck area cancers are limited. It should be mentioned that none of the genes identified in this work are described in the context of the effects of ionizing radiation on the HNSCC.

This study used available data from the TCGA database of both transcriptome and clinical parameters to create a research model to identify potential lncRNAs. In silico analyses showed significant differences in the expression of twenty genes between the groups of patients responding and non-responding to ionizing radiation. The model consisted of patients who received radiation therapy during treatment, and among these patients, a responding and non-responding group was separated based on survival length. Based on comparative analysis, differences were shown for lncRNAs; for the responding group versus the non-responding group, increased expression was observed for C5orf60, C6orf223, MYCNOS, RFPL1S, CASC2, as well as SMCR5 and decreased expression for C10orf55, C2orf27A, C3orf35, C5orf38, TMEM105, HCP5, HHLA3, MEG3, NEAT1, PVT1, SFTA1P, SNHG10, SNHG3, and SNHG7.

In order to verify that the model itself did not affect the results obtained, i.e., specific clinical and pathological parameters, a comparative analysis was performed for the two groups compared. No statistically significant differences in clinical and pathological parameters were observed except for differences in the presence of tumor cells in lymph nodes. This indicates that the established research model appears to be correct. The results were not affected by variables, such as gender, age, tumor stage, and tumor size. It should be noted that TCGA data are often used to create a research model, which is then validated against another database, such as the Gene Expression Omnibus (GEO), patient samples collected at a given center, or cell lines [3,36]. It has been shown that for the genes C10orf55, C3orf35, C5orf38, CASC2, MEG3, MYCNOS, SFTA1P, SNHG3, and TMEM105, there are the greatest differences in expression levels between the group responding and non-responding to radiotherapy. Verification of these data on samples derived from cell lines confirmed this relationship only for genes C10orf55, CASC2, and SFTA1P.

Similar studies have already been conducted, but different results were observed than those presented here [37,38]. Guglas et al. analyzed lncRNA expression in SCC-040, SCC-25, FaDu, and Cal27 cell lines treated with radiation doses of 5, 10, and 20 Gy. It was observed that lncRNA expression is dose-dependant and for a dose of 5 Gy the expression level of HOTAIR, HOXA3as SNHG5, and Zfhx2as were changed; for a dose of 10 Gy, CAR Intergenic 10, Dio3os, HAR1A, Zfhx2as, and HAR1B were changed, and HOXA6as, Zfhx2as, and PTENP1 lncRNAs were changed after a dose of 20 Gy. However, in that study, a defined set of lncRNAs were used for qRT-PCR analysis [37]. In this study, from all available lncRNA transcripts indicated in the RNAseq data, we selected those with the highest differences in expression level between RG and NRG group. Moreover, it should be noted that the radiosensitivity of HNSCC depends on the clinical-pathological features of this heterogeneous group of cancer. For example, ncRNAs encoded by viral particles have modulating ability on the radio-susceptibility in the case of nasopharyngeal carcinoma (NPC) connected with Epstein-Barr virus (EBV) infection [38].

In our opinion, the selection of potential genetic biomarkers based on the TCGA data is a better methodological approach than studies based on cell lines [39]. However, in both cases, this data should be validated in a large number of patients, what is our future goal.

The first indicated lncRNA is C10orf55 (chromosome 10 putative open reading frame 55). The importance of this lncRNA in HNSCC has been demonstrated. Studies have confirmed the close association of C10orf55 with plasminogen activator (PLAU), higher levels of which were unequivocally associated with a worse prognosis of HNSCC. The in vivo and in vitro results suggest an involvement of C10orf55 in tumor cell proliferation and migration [40]. It has also been investigated in acute myeloid leukemia and in complete remission of the disease, following treatment with chemotherapeutics [41]. Dysregulation of this lncRNA after successful treatment with both chemotherapy and radiotherapy offers hope for new diagnostic methods and creates room for more accurate studies. Another cancer in which C10orf55 plays an important role is esophageal adenocarcinoma. A group of researchers has created a compilation of four genes that are targeted by miR-3648 and whose expression closely correlates with the OS time of patients [42]. Unfortunately, based on GSEA analysis, no signaling pathways have been related to the response to ionizing radiation.

The next lncRNA identified in this work is CASC2 (cancer susceptibility 2). Present studies do not indicate an association of CASC2 expression with radiation, although its importance in HNSCC has already been noted. Oral squamous cell carcinoma (OSCC) owes its resistance to chemotherapy to CASC2 [43]. As reported in the literature, the role of this lncRNA in the defense response to tumorigenesis is already known, and its involvement in the apoptotic process has also been noted [44]. CASC2 is also revealed in other cancers, such as gastric, colorectal, and endometrial, always with reduced expression [45].

It should be mentioned that the effect of cisplatin on cells with reduced CASC2 expression in esophageal squamous cell carcinoma was analyzed. It was shown that CASC2 promotes the anti-tumor effect of cisplatin in cancer cells [46]. Based on the results of this study, which also noted CASC2 dysregulation in cells derived from the oral cavity and pharynx, it is suggested that this lncRNA may represent a future biomarker in response to both chemotherapy and radiotherapy. The analysis of changes in signaling pathways performed indicates that patients with low CASC2 expression have increased expression of genes related to the p53 pathway. It was also observed that HNSCC patients responding to radiotherapy have an increased expression of this lncRNA. DNA damage is known to stabilize p53 in part through the DNA damage signaling pathway, which involves sensory kinases, including ATM and ATR, and effector kinases, such as Chk1/2 and Wee1, which lead to post-transcriptional regulation of various genes involved in DNA repair, cell cycle control, apoptosis, and aging [47]. The results obtained and the literature data indicate a role for lncRNA CASC2 in the radiation response and its likely importance in the p53 pathway, which should be further analyzed.

The last lncRNA whose expression changes under the influence of ionizing radiation is SFTA1P (surfactant associated 1 pseudogene). As numerous studies have shown, SFTA1P is closely associated with lung diseases [48]. In cell lines of non-small cell lung cancer, it is transcriptionally activated, which is responsible for the inhibition of cell proliferation or induction of apoptosis [48]. However, in 2020 and 2021, two studies were conducted, demonstrating its importance also in HNSCC [49,50]. SFTA1P has potential prognostic significance and can be used to assess survival outcomes [50]. Based on an in vitro model, a change in the expression of SFTA1P after treatment of lung squamous cell carcinoma cells with cisplatin. It was found that SFTA1P, due to its correlation with the response to cisplatin, would be in the future a good biomarker in predicting response to chemotherapy [50]. Analysis of signaling pathways showed that in a group of patients with high lncRNA expression SFTA1P showed enhanced expression of genes related to the epithelial-to-mesenchymal transition. It should be noted that the expression level of this lncRNA is also high in the group of patients undergoing radiotherapy and with a short survival period (non-responders). It is known that cells that undergo a change in phenotype from epithelial to mesenchymal are characterized by greater aggressiveness, ability to metastasize, and resistance to both ionizing radiation and exposure to chemotherapeutics. This resistance is closely related to the alteration of the cellular program [51]. SFTA1P appears to be one of the lncRNAs associated with acquiring a more malignant cellular phenotype.

There is no literature data in relation to changes in the expression of this lncRNA under the influence of ionizing radiation, and the demonstrated potential importance of this lncRNA in the presented results of this work provides new opportunities for understanding the role of this lncRNA in HNSCC.

We also checked the immune profile of HNSCC patients depending on the expression levels of C10orf55, C3orf35, C5orf38, CASC2, MEG3, MYCNOS, SFTA1P, SNHG3, and TMEM105 lncRNAs. We indicated that with higher levels of C5orf38, SNHG3 and TMEM105 patients displayed lower levels of immune score and lymphocyte infiltration signature score. Moreover, higher levels of C5orf38, SNHG3, and TMEM105 were characteristic for patients from non-responders’ groups (NRG), which displayed shorter OS time. Chen et al., based on the analysis of lncRNAs in glioma patients, indicated that C5orf38 (chromosome 5 open reading frame 38) is one of the necroptosis-related lncRNAs and was one of the protective factors [52]. It is known that SNHG3 (small nucleolar RNA host gene 3) regulates EZH2, which in turn influences the promoter methylation of KLF2 (Krüppel-like Factor 2) and p21 genes. KLF2 is a zinc-finger transcription factor responsible for activation of CD4+ T cells [53]. Unfortunately, there is no literature information about the potential role and significance of TMEM105 lncRNA (TMEM105 long non-coding RNA) in cancer immunology, and it is difficult to discuss the observed results for HNSCC patients.

Similar results were in the case of MEG3 and MYCNOS. MEG3 is downregulated in patients responding to radiotherapy, and in these patients, a lower level of MEG3 is associated with a higher level of lymphocyte infiltration signature score. Xu et al. investigated the role of MEG3 (maternally expressed gene 3) as a prognostic factor and its immune-related role in gliomas. They observed that lower levels of MEG3 were associated with shorter patients’ survival. In low-grade glioma, MEG3 was negatively correlated with infiltrating B cells, CD8+ T cells, CD4+ T cells, macrophages, neutrophils, and dendritic cells, but in the case of glioblastoma multiforme, MEG3 was positively correlated with infiltrating CD8+ T cells and negatively correlated with infiltrating dendritic cells [54]. The immunomodulatory role of MEG3 was also observed by Wang et al., and they showed that MEG3 was downregulated in CD4+ T cells derived from aplastic anemia patients. They proposed that regulation of CD4+ T cell activation depended on MEG3, which in turn regulated miR-23a expression level and finally influenced TIGIT (T cell immunoreceptor with Ig and ITIM domains) [55].

The last lncRNA associated with the immune profile was MYCNOS (MYCN opposite strand). Higher levels of lymphocytes were associated with MYCNOS lncRNAs, which are upregulated in the responding group (RG) and display longer survival in comparison to the non-responders’ group (NRG). It is known that MYCNOS lncRNA regulates the expression of MYCN by binding to its promoter and influencing cancer cell phenotype [56], but its role in the regulation of immune cells has not been indicated.

C5orf38, SNHG3, TMEM105, MEG3, and MYCNOS lncRNAs seem to be potential biomarkers describing the immune profile of HNSCC patients in response to radiotherapy. However, it should be verified based on more data from in vitro and in vivo models.

In conclusion, ionizing radiation is certainly an important factor affecting the expression of long non-coding RNAs in head and neck cancers. For their full understanding, however, more extensive analyses need to be conducted using more samples tested, both in vitro and in vivo. The small number of relevant genes obtained in this study may be due to the juxtaposition of results derived from only six cell lines with results from one hundred and fifty patients. It is also worth noting that cell lines do not always reflect the cellular phenotype of cancer patients, which is why it is important to conduct further research.

In light of the above evidence, it can be suggested that the expression levels of C10orf55, CASC2, and SFTA1P in the future may be a prognostic factor in assessing the patient’s response to radiotherapy.

## Figures and Tables

**Table 1:** Primer sequences used in the qRT-PCR reaction.

lncRNA	Forward Primer (5′-3′)	Right Primer (5′-3′)	Ref:
C10orf55	ATTCGGGAGGAGGCTTCATCA	TGAGAACTAGATACGAACAGGGT	[29]
C3orf35	AAGAGGTTATTGTGCGCCCG	ATTAGCCCGCCTTCCTCTGT	[30]
C5orf38	CTGCTGCCTGTTACTAAT	CAATGACGAGTGTTAAGTT	[31]
CASC2	GGCTCACAAAGCCTAGGTTA	CCTTGGATATTTCCAAGAGC	[32]
MEG3	CTGCCCATCTACACCTCACG	CTCTCCGCCGTCTGCGCTAGGGGCT	[33]
MYCNOS	TCCGACAGCTCAAACACAGAC	CCAGCTTTGCAGCCTTCTC	[34]
SFTA1P	CATTCCAGGTGGGCTTTCA	TCCCTTGTTTGGCTTACTCG	[35]
SNHG3	AATCCAGTCTCATTCAGTAA	GCGTCCTAATCATTCAATC	[31]
TMEM105	TGGCAGCAGGGATAACAG	TGAGCAACAGAGCAAGACT	[31]
GAPDH	CCACTCCTCCACCTTTGACG	CCACCACCCTGTTGCTGTAG	[31]

## Data Availability

The data presented in this study are openly available at GEO and TCGA databases, and its analysis and results presented in this work do not violate any copyrights.

## Supplementary Materials

The following are available online at https://www.mdpi.com/article/10.3390/jpm12101696/s1, Table S1: Patients included in the study with division to responders group (RG) and non-responders group (NRG); Table S2: Main clinical and pathological parameters describing RG and NRG groups; *p* < 0.05 considered as statistically significant; Table S3: lncRNA expression profile in responders group (RG) and non-responders group (NRG) of patients to radiotherapy; Table S4: The area under the ROC Curve (*AUC*) to discriminate between the RG and NRG groups; *p* < 0.05 considered as statistically significant; Table S5: Mean expression level of lncRNAs in healthy and HNSCC patients; *p* < 0.05 considered as statistically significant; Table S6: GSEA analysis of genes enriched in the set of 150 HNSCC patients (RG and NRG groups) depending on expression level of specified lncRNA (high or low groups divided based on median expression of lncRNA); only the gene sets with nominal *p* < 0.05 were presented; *NES*-normalized enrichment score, *FDR q*-value-false discovery rate, SIZE-number of enriched genes in a specified process; and further analysis of genes included in GSEA results (*FDR* < 0.27 and *p* < 0.05); Table S7: Genes enriched in the group of patients with higher levels of SFTA1 and SNHG3 lncRNAs and lower levels of C3orf35, CASC2, and MEG3, and connected with ionization radiation response; Table S8: Analysis of infiltration of immune cells into tumor tissues and infer tumor purity depending on the lncRNA expression level; *p* < 0.05 considered significant.

## References

[1] Mandal R., Şenbabaoğlu Y., Desrichard A., Havel J.J., Dalin M.G., Riaz N., Lee K.W., Ganly I., Hakimi A.A., Chan T.A. (2016). The head and neck cancer immune landscape and its immunotherapeutic implications. JCI Insight.

[2] Conway D.I., Hashibe M., Boffetta P., Wunsch-Filho V., Muscat J., La Vecchia C., Winn D.M. (2009). Enhancing epidemiologic research on head and neck cancer: INHANCE—The international head and neck cancer epidemiology consortium. Oral Oncol..

[3] Shen Y., Liu J., Zhang L., Dong S., Zhang J., Liu Y., Zhou H., Dong W. (2019). Identification of Potential Biomarkers and Survival Analysis for Head and Neck Squamous Cell Carcinoma Using Bioinformatics Strategy: A Study Based on TCGA and GEO Datasets. BioMed Res. Int..

[4] Jethwa A.R., Khariwala S.S. (2017). Tobacco-related carcinogenesis in head and neck cancer. Cancer Metastasis Rev..

[5] Vokes E.E., Agrawal N., Seiwert T.Y. (2015). HPV-Associated Head and Neck Cancer. J. Natl. Cancer Inst..

[6] Specenier P., Vermorken J.B. (2018). Optimizing treatments for recurrent or metastatic head and neck squamous cell carcinoma. Expert Rev. Anticancer Ther..

[7] Johnson D.E., Burtness B., Leemans C.R., Lui V.W.Y., Bauman J.E., Grandis J.R. (2020). Head and neck squamous cell carcinoma. Nat. Rev. Dis. Primers.

[8] Cramer J.D., Burtness B., Le Q.T., Ferris R.L. (2019). The changing therapeutic landscape of head and neck cancer. Nat. Rev. Clin. Oncol..

[9] Alsahafi E., Begg K., Amelio I., Raulf N., Lucarelli P., Sauter T., Tavassoli M. (2019). Clinical update on head and neck cancer: Molecular biology and ongoing challenges. Cell Death Dis..

[10] O’Rorke M.A., Ellison M.V., Murray L.J., Moran M., James J., Anderson L.A. (2012). Human papillomavirus related head and neck cancer survival: A systematic review and meta-analysis. Oral Oncol..

[11] Ang K.K., Harris J., Wheeler R., Weber R., Rosenthal D.I., Nguyen-Tân P.F., Westra W.H., Chung C.H., Jordan R.C., Lu C. (2010). Human papillomavirus and survival of patients with oropharyngeal cancer. N. Engl. J. Med..

[12] Zhou C., Parsons J.L. (2020). The radiobiology of HPV-positive and HPV-negative head and neck squamous cell carcinoma. Expert Rev. Mol. Med..

[13] Huang R.X., Zhou P.K. (2020). DNA damage response signaling pathways and targets for radiotherapy sensitization in cancer. Signal Transduct. Target. Ther..

[14] Baskar R., Dai J., Wenlong N., Yeo R., Yeoh K.W. (2014). Biological response of cancer cells to radiation treatment. Front. Mol. Biosci..

[15] Feller G., Khammissa R.A.G., Nemutandani M.S., Feller L. (2021). Biological consequences of cancer radiotherapy in the context of oral squamous cell carcinoma. Head Face Med..

[16] Wang J., Wang H., Qian H. (2018). Biological effects of radiation on cancer cells. Mil. Med. Res..

[17] Han P.B., Ji X.J., Zhang M., Gao L.Y. (2018). Upregulation of lncRNA LINC00473 promotes radioresistance of HNSCC cells through activating Wnt/β-catenin signaling pathway. Eur. Rev. Med. Pharmacol. Sci..

[18] Chen L.L. (2016). Linking Long Noncoding RNA Localization and Function. Trends Biochem. Sci..

[19] Kozłowska J., Kolenda T., Poter P., Sobocińska J., Guglas K., Stasiak M., Bliźniak R., Teresiak A., Lamperska K. (2021). Long Intergenic Non-Coding RNAs in HNSCC: From “Junk DNA” to Important Prognostic Factor. Cancers.

[20] Mercer T.R., Dinger M.E., Mattick J.S. (2009). Long non-coding RNAs: Insights into functions. Nat. Rev. Genet..

[21] Ransohoff J.D., Wei Y., Khavari P.A. (2018). The functions and unique features of long intergenic non-coding RNA. Nat. Rev. Mol. Cell Biol..

[22] Smith K.N., Miller S.C., Varani G., Calabrese J.M., Magnuson T. (2019). Multimodal Long Noncoding RNA Interaction Networks: Control Panels for Cell Fate Specification. Genetics.

[23] Wang W., Min L., Qiu X., Wu X., Liu C., Ma J., Zhang D., Zhu L. (2021). Biological Function of Long Non-coding RNA (LncRNA) Xist. Front. Cell Dev. Biol..

[24] Yoshihara K., Shahmoradgoli M., Martínez E., Vegesna R., Kim H., Torres-Garcia W., Treviño V., Shen H., Laird P.W., Levine D.A. (2013). Inferring tumour purity and stromal and immune cell admixture from expression data. Nat. Commun..

[25] Thorsson V., Gibbs D.L., Brown S.D., Wolf D., Bortone D.S., Ou Yang T.H., Porta-Pardo E., Gao G.F., Plaisier C.L., Eddy J.A. (2018). The Immune Landscape of Cancer. Immunity.

[26] Warde-Farley D., Donaldson S.L., Comes O., Zuberi K., Badrawi R., Chao P., Franz M., Grouios C., Kazi F., Lopes C.T. (2010). The Gene MANIA prediction server: Biological network integration for gene prioritization and predicting gene function. Nucleic Acids Res..

[27] Lindell Jonsson E., Erngren I., Engskog M., Haglöf J., Arvidsson T., Hedeland M., Petterson C., Laurell G., Nestor M. (2019). Exploring Radiation Response in Two Head and Neck Squamous Carcinoma Cell Lines Through Metabolic Profiling. Front. Oncol..

[28] Guglas K., Kolenda T., Stasiak M., Kopczyńska M., Teresiak A., Ibbs M., Bliźniak R., Lamperska K. (2020). YRNAs: New Insights and Potential Novel Approach in Head and Neck Squamous Cell Carcinoma. Cells.

[29] Chen G., Sun J., Xie M., Yu S., Tang Q., Chen L. (2021). PLAU Promotes Cell Proliferation and Epithelial-Mesenchymal Transition in Head and Neck Squamous Cell Carcinoma. Front. Genet..

[30] Shi Y., Ren J., Zhuang Z., Zhang W., Wang Z., Liu Y., Li J., Liang T., He R., Wang K. (2020). Comprehensive Analysis of a ceRNA Network Identifies lncRC3orf35 Associated with Poor Prognosis in Osteosarcoma. BioMed Res. Int..

[31] http://www.oligoarchitect.com/AlternatePrimers.jsp.

[32] Tao L., Tian P., Yang L., Guo X. (2020). lncRNA CASC2 Enhances 131I Sensitivity in Papillary Thyroid Cancer by Sponging miR-155. BioMed Res. Int..

[33] Wu M., Huang Y., Chen T., Wang W., Yang S., Ye Z., Xi X. (2019). LncRNA MEG3 inhibits the progression of prostate cancer by modulating miR-9-5p/QKI-5 axis. J. Cell Mol. Med..

[34] Yu J., Ou Z., Lei Y., Chen L., Su Q., Zhang K. (2020). LncRNA MYCNOS facilitates proliferation and invasion in hepatocellular carcinoma by regulating miR-340. Hum. Cell.

[35] Du D., Shen X., Zhang Y., Yin L., Pu Y., Liang G. (2020). Expression of long non-coding RNA SFTA1P and its function in non-small cell lung cancer. Pathol. Res. Pract..

[36] Yu D., Pan M., Li Y., Lu T., Wang Z., Liu C., Hu G. (2022). RNA N6-methyladenosine reader IGF2BP2 promotes lymphatic metastasis and epithelial-mesenchymal transition of head and neck squamous carcinoma cells via stabilizing slug mRNA in an m6A-dependent manner. J. Exp. Clin. Cancer Res..

[37] Guglas K., Kolenda T., Teresiak A., Kopczyńska M., Łasińska I., Mackiewicz J., Mackiewicz A., Lamperska K. (2018). lncRNA Expression after Irradiation and Chemoexposure of HNSCC Cell Lines. Noncoding RNA.

[38] Lei F., Lei T., Huang Y., Yang M., Liao M., Huang W. (2020). Radio-Susceptibility of Nasopharyngeal Carcinoma: Focus on Epstein- Barr Virus, MicroRNAs, Long Non-Coding RNAs and Circular RNAs. Curr. Mol. Pharmacol..

[39] Li J., Li Y., Wu X., Li Y. (2019). Identification and validation of potential long non-coding RNA biomarkers in predicting survival of patients with head and neck squamous cell carcinoma. Oncol. Lett..

[40] Walker C.J., Mrózek K., Ozer H.G., Nicolet D., Kohlschmidt J., Papaioannou D., Genutis L.K., Bill M., Powell B.L., Uy G.L. (2021). Gene expression signature predicts relapse in adult patients with cytogenetically normal acute myeloid leukemia. Blood Adv..

[41] Zhang D., Yin H., Bauer T.L., Rogers M.P., Velotta J.B., Morgan C.T., Du W., Xu P., Qian X. (2021). Development of a novel miR-3648-related gene signature as a prognostic biomarker in esophageal adenocarcinoma. Ann. Transl. Med..

[42] O’Leary V.B., Ovsepian S.V., Carrascosa L.G., Buske F.A., Radulovic V., Niyazi M., Moertl S., Trau M., Atkinson M.J., Anastasov N. (2015). PARTICLE, a Triplex-Forming Long ncRNA, Regulates Locus-Specific Methylation in Response to Low-Dose Irradiation. Cell Rep..

[43] https://www.ncbi.nlm.nih.gov/gene/255082.

[44] Zhang C., Cao W., Wang J., Liu J., Liu J., Wu H., Li S., Zhang C. (2020). A prognostic long noncoding RNA-associated competing endogenous RNA network in head and neck squamous cell carcinoma. PeerJ.

[45] Zhu D., Yu Y., Qi Y., Wu K., Liu D., Yang Y., Zhang C., Zhao S. (2019). Long Non-coding RNA CASC2 Enhances the Antitumor Activity of Cisplatin Through Suppressing the Akt Pathway by Inhibition of miR-181a in Esophageal Squamous Cell Carcinoma Cells. Front. Oncol..

[46] Lindemann A., Takahashi H., Patel A.A., Osman A.A., Myers J.N. (2018). Targeting the DNA Damage Response in OSCC with TP53 Mutations. J. Dent. Res..

[47] Nathan N., Berdah L., Delestrain C., Sileo C., Clement A. (2020). Interstitial lung diseases in children. Presse Med..

[48] Zhu B., Finch-Edmondson M., Leong K.W., Zhang X., Lin Q.X.X., Lee Y., Ng W.T., Guo H., Wan Y., Sudol M. (2021). LncRNA SFTA1P mediates positive feedback regulation of the Hippo-YAP/TAZ signaling pathway in non-small cell lung cancer. Cell Death Discov..

[49] Jiang W., Song Y., Zhong Z., Gao J., Meng X. (2021). Ferroptosis-Related Long Non-Coding RNA Signature Contributes to the Prediction of Prognosis Outcomes in Head and Neck Squamous Cell Carcinomas. Front. Genet..

[50] Li L., Yin J.Y., He F.Z., Huang M.S., Zhu T., Gao Y.F., Chen Y.X., Zhou D.B., Chen X., Sun L.Q. (2017). Long noncoding RNA SFTA1P promoted apoptosis and increased cisplatin chemosensitivity via regulating the hnRNP-U-GADD45A axis in lung squamous cell carcinoma. Oncotarget.

[51] Zhou S., Zhang M., Zhou C., Wang W., Yang H., Ye W. (2020). The role of epithelial-mesenchymal transition in regulating radioresistance. Crit. Rev. Oncol. Hematol..

[52] Chen D., Dou C., Liu H., Xu B., Hu B., Kuang L., Yao J., Zhao Y., Yu S., Li Y. (2022). Comprehensive analysis: Necroptosis-related lncRNAs can effectively predict the prognosis of glioma patients. Front. Oncol..

[53] Zimta A.A., Tigu A.B., Braicu C., Stefan C., Ionescu C., Berindan-Neagoe I. (2020). An Emerging Class of Long Non-coding RNA With Oncogenic Role Arises From the snoRNA Host Genes. Front. Oncol..

[54] Xu X., Zhong Z., Shao Y., Yi Y. (2021). Prognostic Value of *MEG3* and Its Correlation with Immune Infiltrates in Gliomas. Front. Genet..

[55] Wang J., Liu X., Hao C., Lu Y., Duan X., Liang R., Gao G., Zhang T. (2019). MEG3 modulates TIGIT expression and CD4 + T cell activation through absorbing miR-23a. Mol. Cell Biochem..

[56] Suenaga Y., Nakatani K., Nakagawara A. (2020). De novo evolved gene product NCYM in the pathogenesis and clinical outcome of human neuroblastomas and other cancers. Jpn. J. Clin. Oncol..

