# Supplementary material for: C10orf55, CASC2, and SFTA1P lncRNAs Are Potential Biomarkers to Assess Radiation Therapy Response in Head and Neck Cancers
Source: J Pers Med. 2022 Oct 11;12(10):1696. doi: 10.3390/jpm12101696 (PMC9605465; doi:10.3390/jpm12101696)
Supplement: Supplementary file 1 [file jpm-12-01696-s001.zip › jpm-1920533-supplementary.pdf]

Supplementary materials:

Table S1. Patients included in the study with division to responders group (RG) and non-responders group (NRG).

Responders Group (RG)			No-Responders Group (NRG)		
Sample ID	OS time (days)	OS event (1-dead; 0-alive)	Sample ID	OS time (days)	OS event (1-dead; 0-alive)
TCGA-BA-5152-01	1288	0	TCGA-BA-4074-01	462	1
TCGA-BA-5558-01	1995	0	TCGA-BA-4076-01	415	1
TCGA-BA-7269-01	1273	0	TCGA-BA-4078-01	276	1
TCGA-BB-4223-01	3221	0	TCGA-BA-5149-01	806	1
TCGA-BB-7862-01	1117	0	TCGA-BA-6868-01	472	1
TCGA-BB-7864-01	1527	0	TCGA-BA-6870-01	451	1
TCGA-BB-7866-01	1368	0	TCGA-BA-6872-01	384	1
TCGA-BB-7870-01	2016	0	TCGA-BA-A6DD-01	173	1
TCGA-BB-7872-01	1168	0	TCGA-BA-A6DF-01	238	1
TCGA-BB-8596-01	2161	0	TCGA-BA-A6DG-01	69	1
TCGA-CN-4727-01	1560	0	TCGA-BA-A6DI-01	336	1
TCGA-CN-4728-01	1724	0	TCGA-BA-A6DJ-01	407	1
TCGA-CN-4734-01	1690	0	TCGA-BB-A5HY-01	321	1
TCGA-CN-4735-01	1737	0	TCGA-BB-A6UO-01	268	1
TCGA-CN-5360-01	2169	0	TCGA-CN-4742-01	397	1
TCGA-CN-5373-01	1584	0	TCGA-CN-5359-01	377	1
TCGA-CN-6010-01	1523	0	TCGA-CN-5364-01	493	1
TCGA-CN-6012-01	1460	0	TCGA-CN-5366-01	360	1
TCGA-CN-6016-01	1443	0	TCGA-CN-5367-01	352	1
TCGA-CN-6023-01	1584	0	TCGA-CN-6013-01	727	1
TCGA-CN-6992-01	1066	0	TCGA-CN-6017-01	853	1
TCGA-CQ-5323-01	1466	0	TCGA-CN-6020-01	205	1
TCGA-CQ-5327-01	1660	0	TCGA-CN-6996-01	530	1
TCGA-CQ-5330-01	1897	0	TCGA-CN-6998-01	357	1
TCGA-CQ-6218-01	1253	0	TCGA-CN-A498-01	773	1
TCGA-CQ-6222-01	2016	0	TCGA-CN-A49A-01	526	1
TCGA-CQ-6224-01	1721	0	TCGA-CN-A63W-01	377	1
TCGA-CQ-7064-01	1973	0	TCGA-CQ-5326-01	89	1
TCGA-CQ-7071-01	1311	0	TCGA-CQ-6228-01	456	1
TCGA-CQ-7072-01	2359	0	TCGA-CQ-A4CH-01	379	1
TCGA-CR-5243-01	2562	0	TCGA-CR-6474-01	564	1
TCGA-CR-5249-01	1152	0	TCGA-CR-6478-01	183	1
TCGA-CR-6467-01	1777	0	TCGA-CR-6493-01	282	1
TCGA-CR-6470-01	1521	0	TCGA-CR-7380-01	606	1
TCGA-CR-6472-01	1050	0	TCGA-CV-5431-01	522	1
TCGA-CR-6473-01	1125	0	TCGA-CV-5439-01	546	1
TCGA-CR-7365-01	1191	0	TCGA-CV-5966-01	545	1

TCGA-CR-7367-01	1440	0	TCGA-CV-6936-01	166	1
TCGA-CR-7368-01	1245	0	TCGA-CV-6941-01	342	1
TCGA-CR-7386-01	1430	0	TCGA-CV-6950-01	459	1
TCGA-CR-7390-01	1508	0	TCGA-CV-6952-01	185	1
TCGA-CR-7392-01	1425	0	TCGA-CV-6962-01	126	1
TCGA-CR-7394-01	1346	0	TCGA-CV-7252-01	151	1
TCGA-CR-7404-01	1472	0	TCGA-CV-7418-01	789	1
TCGA-CV-5430-01	4241	0	TCGA-CV-7429-01	107	1
TCGA-CV-5440-01	3270	0	TCGA-CV-7433-01	601	1
TCGA-CV-5441-01	2886	0	TCGA-CV-A45X-01	198	1
TCGA-CV-5973-01	2641	0	TCGA-CV-A468-01	464	1
TCGA-CV-5977-01	1840	0	TCGA-CV-A6JD-01	182	1
TCGA-CV-5979-01	1315	0	TCGA-CV-A6JO-01	197	1
TCGA-CV-7090-01	5252	0	TCGA-CV-A6K2-01	317	1
TCGA-CV-7235-01	2347	0	TCGA-D6-6515-01	403	1
TCGA-CV-7238-01	2727	0	TCGA-DQ-5631-01	548	1
TCGA-CV-7261-01	1512	0	TCGA-HD-8224-01	446	1
TCGA-CV-A45R-01	5480	0	TCGA-IQ-A61K-01	161	1
TCGA-CX-A4AQ-01	1555	0	TCGA-IQ-A61O-01	421	1
TCGA-DQ-5624-01	1778	0	TCGA-KU-A66S-01	406	1
TCGA-DQ-7589-01	1409	0	TCGA-KU-A6H8-01	327	1
TCGA-DQ-7590-01	1413	0	TCGA-P3-A5Q6-01	480	1
TCGA-DQ-7592-01	1143	0	TCGA-P3-A5QF-01	330	1
TCGA-DQ-7593-01	1224	0	TCGA-P3-A6T3-01	577	1
TCGA-DQ-7594-01	1218	0	TCGA-P3-A6T6-01	395	1
TCGA-DQ-7595-01	1190	0	TCGA-P3-A6T7-01	487	1
TCGA-DQ-7596-01	1265	0	TCGA-QK-A6IG-01	222	1
TCGA-F7-7848-01	1131	0	TCGA-QK-A6II-01	284	1
TCGA-IQ-A61E-01	1147	0	TCGA-QK-A8Z9-01	449	1
TCGA-P3-A5QA-01	2182	0	TCGA-QK-A8ZA-01	371	1
TCGA-P3-A5QE-01	1559	0	TCGA-RS-A6TO-01	387	1
TCGA-P3-A6SW-01	1120	0	TCGA-T2-A6WX-01	209	1
TCGA-P3-A6T2-01	2298	0	TCGA-T2-A6WZ-01	484	1
TCGA-UF-A71B-01	1506	0	TCGA-UF-A7JC-01	546	1
TCGA-UF-A71D-01	1461	0	TCGA-UF-A7JD-01	739	1
TCGA-UF-A7J9-01	1358	0	TCGA-UF-A7JK-01	424	1
TCGA-UF-A7JA-01	2265	0	TCGA-UF-A7JO-01	631	1
TCGA-UF-A7JF-01	1686	0	TCGA-UF-A7JS-01	680	1

Table S2. Main clinical and pathological parameters describing RG and NRG groups; $p < 0.05$ considered as statistically significant.

Parameter	Group	RG (cases)	NRG (cases)	p-value
Gender	Male	60	55	0.3344
	Female	15	20	
Alcohol	Yes	61	57	0.4253
	no	14	18	
Smoking	Yes	27	34	0.2424
	No/ex	47	40	
Cancer stage	I. II	9	6	0.4142
	III. IV	66	69	
T-stage	T1+T2	21	12	0.0761
	T3+T4	54	63	
N-stage	N0	24	25	0.8621
	N1/2/3	50	48	
Grade	G1+G2	45	55	0.1241
	G3+G4	27	19	
Perineural invasion	Positive	30	37	0.0816
	Negative	26	16	
Lymph node neck dissection	Positive	60	66	0.4253
	negative	15	9	
HPV p16 status	Positive	7	2	0.1982
	Negative	15	13	
Targeted molecular therapy	YES	37	39	0.3813
	NO	23	34	
Additional pharmaceutical therapy	YES	3	14	0.5274
	NO	6	17	
Localization	Buccal Mucosa	4	2	0.1231
	Floor of mouth	6	15	
	Tonsil	10	2	
	Oral Tongue	18	20	
	Larynx	20	13	

	Oral Cavity	7	8	
	HYPOPHARYNX	2	2	
	Base of tongue	4	4	
	Alveolar Ridge	3	6	
	HARD PALATE	1	0	
	Lip	0	1	
	Oropharynx	0	2	

Table S3. lncRNA expression profile in responders group (RG) and non-responders group (NRG) of patients to radiotherapy.

lncRNA	Group	Minimum	25% Percentile	Median	75% Percentile	Maximum	Range	Mean	Std. Deviation	Std. Error of Mean
C10orf55	RG	2.137	4.82	5.667	6.18	7.671	5.534	5.431	1.106	0.1295
	NRG	3.374	5.52	6.287	6.528	08.07	4.696	06.07	0.8949	0.1047
C2orf27A	RG	1.729	3.315	3.884	4.875	7.594	5.865	4.121	1.077	0.126
	NRG	1.941	3.815	4.395	4.957	8.124	6.183	4.524	1.215	0.1422
C3orf35	RG	0	1.256	1.68	2.098	3.255	3.255	1.677	0.7767	0.09091
	NRG	0	1.396	2.069	2.615	4.356	4.356	2.081	0.944	0.1105
C5orf38	RG	0.3389	4.195	5.174	6.184	9.993	9.654	5.067	1.913	0.2223
	NRG	0	5.325	6.202	6.896	10.47	10.47	6.079	1.556	0.1809
C5orf60	RG	0	0	0.5158	0.9496	8.234	8.234	0.6538	1.082	0.1258
	NRG	0	0	0	0.6096	4.711	4.711	0.4668	0.8302	0.09651
C6orf223	RG	0.8317	2.518	4.59	6.752	8.969	8.137	4.681	2.307	0.2682
	NRG	0	2.156	2.859	5.757	9.599	9.599	3.832	2.322	0.27
CASC2	RG	2.338	3.701	4.184	4.851	8.234	5.896	4.282	0.9644	0.1121
	NRG	1.241	3.421	3.839	4.341	5.73	4.489	3.874	0.8408	0.09774
HCP5	RG	6.603	9.409	10.43	10.97	12.05	5.449	10.2	01.06	0.124
	NRG	7.286	10.12	10.83	11.21	12.32	5.033	10.59	1.013	0.1185

	RG	4.482	5.967	6.468	6.994	9.606	5.124	6.48	0.9244	0.1082
**HHLA3**	NRG	4.861	6.294	6.826	7.289	8.909	4.047	6.814	0.8444	0.09883
	RG	0.677	3.465	4.693	5.656	8.576	7.899	4.674	1.676	0.1962
**MEG3**	NRG	1.107	4.363	5.324	6.839	9.584	8.477	5.497	1.75	0.2048
	RG	0	0	0	0.6541	1.873	1.873	0.4144	0.5473	0.06405
**MYCNOS**	NRG	0	0	0	0	1.319	1.319	0.135	0.3246	0.03799
	RG	9.566	11.36	12.3	12.91	14.97	5.403	12.07	1.052	0.1231
**NEAT1**	NRG	9.572	11.66	12.58	13.43	14.83	5.253	12.48	1.304	0.1527
	RG	4.487	6.017	6.73	7.349	10.21	5.726	6.719	1.063	0.1244
**PVT1**	NRG	5.225	6.356	6.943	7.718	9.171	3.946	7.079	0.9052	0.1059
	RG	0	1.059	1.888	3.164	7.916	7.916	2.527	1.98	0.2317
**RFPL1S**	NRG	0	0.6681	1.369	2.601	8.626	8.626	1.817	1.617	0.1893
	RG	0	0.8037	1.765	3.569	8.88	8.88	2.259	1.878	0.2198
**SFTA1P**	NRG	0	1.728	2.797	4.637	8.802	8.802	3.266	02.04	0.2388
	RG	0	0	0.5542	1.091	2.239	2.239	0.6252	0.6377	0.07464
**SMCR5**	NRG	0	0	0	0.6897	3.097	3.097	0.4221	0.6414	0.07507
	RG	3.174	4.868	5.621	6.265	7.343	4.169	5.56	1.018	0.1191
**SNHG10**	NRG	3.542	5.129	6.024	6.883	9.273	5.731	6.005	1.11	0.1299
	RG	3.346	4.748	5.687	6.576	8.036	4.689	5.672	1.178	0.1378
**SNHG3**	NRG	3.764	5.465	6.207	7.113	9.377	5.612	6.24	1.163	0.1361
	RG	5.89	7.755	8.412	9.049	11.31	5.424	8.466	01.02	0.1194
**SNHG7**	NRG	6.424	8.016	8.731	9.522	12.18	5.757	8.826	1.115	0.1305
	RG	0	1.976	2.92	4.098	6.634	6.634	3.013	1.566	0.1833
**TMEM105**	NRG	0	3.115	3.938	5.079	7.105	7.105	3.951	1.614	0.1889

Table S4. Area under the ROC Curve (AUC) to discriminate between the RG and NRG groups; $p < 0.05$ considered as statistically significant.

lncRNA	AUC	p-value	Std. Error
C5orf60	0.5898	0.0592	0.04706
SNHG7	0.5954	0.0466	0.04705

C2orf27A	0.5996	0.0377	0.04735
SMCR5	0.6027	0.0321	0.04697
PVT1	0.6042	0.0297	0.04663
RFPL1S	0.6056	0.0277	0.0466
C6orf223	0.6063	0.0256	0.04684
NEAT1	0.6078	0.0245	0.04703
SNHG10	0.6082	0.024	0.0465
HCP5	0.6181	0.0137	0.04637
HHLA3	0.6191	0.013	0.04653
CASC2	0.6211	0.011	0.04588
C3orf35	0.6239	0.0097	0.04666
SNHG3	0.6292	0.007	0.04587
MEG3	0.6319	0.0059	0.04598
MYCNOS	0.6544	0.0013	0.04554
SFTA1P	0.6577	0.001	0.04509
TMEM105	0.672	0.0003	0.04492
C5orf38	0.6777	0.0002	0.0442
C10orf55	0.6784	0.0002	0.04433

Table S5. Mean expression level of lncRNAs in healthy and HNSCC patients; $p < 0.05$ considered as statistically significant.

lncRNA	Healthy (Mean expression)	Patients (Mean expression)	p-val
SNHG3	5.193	5.92	0.0001
C5orf60	0.1832	0.5528	0.0002
PVT1	6.415	6.875	0.0013
C6orf223	3.461	4.408	0.0135
TMEM105	2.843	3.323	0.0357
MYCNOS	0.4133	0.2998	0.0472
NEAT1	11.92	12.26	0.0841
MEG3	5.534	4.946	0.1824
SMCR5	0.4419	0.4953	0.5756
SNHG7	8.77	8.679	0.5826
C3orf35	1.826	1.772	0.6029
CASC2	4.251	4.034	0.7752

HHLA3	6.592	6.621	0.8326
RFPL1S	1.936	2.229	0.9121
C10orf55	4.283	5.653	< 0.0001
C2orf27A	3.511	4.257	< 0.0001
C5orf38	4.518	5.583	< 0.0001
HCP5	9.294	10.4	< 0.0001
SFTA1P	1.04	2.591	< 0.0001
SNHG10	5.099	5.849	< 0.0001

Table S6. GSEA analysis of genes enriched in the set of 150 HNSCC patients (RG and NRG groups) depending on expression level of specified lncRNA (high or low groups divided based on median expression of lncRNA); only the gene sets with nominal $p < 0.05$ were presented; *NES*—normalized enrichment score, *FDR q-value*—false discovery rate, SIZE—number of enriched genes in a specified process; and further analysis of genes included in GSEA results (*FDR* < 0.27 and *p* < 0.05).

**lncRNA**	**Process/Pathway**	**SIZE (number of genes enriched in process/pathway)**	**FDR q-value**	**p-value**	**Enrichment in group of patients with expression level of lncRNA**
SFTA1	EPITHELIAL MESENCHYMAL TRANSITION	194	0.12196739	0.0	high
SNHG3	DNA REPAIR	139	0.14401889	0.040733196	high
SFTA1	ANGIOGENESIS	36	0.22596863	0.001996008	high
SFTA1	COAGULATION	136	0.25195256	0.008	high
MEG3	MITOTIC SPINDLE	196	0.029923137	0.003944773	low
CASC2	P53 PATHWAY	190	0.030040383	0.0	low
C3orf35	MITOTIC SPINDLE	196	0.087503836	0.018292682	low
MEG3	G2M CHECKPOINT	184	0.10796102	0.036734693	low
C3orf35	ANDROGEN RESPONSE	96	0.25371048	0.01934236	low
C3orf35	UV RESPONSE DN	137	0.26422462	0.014553014	low

Table S7. Genes enriched in the group of patients with higher levels of SFTA1 and SNHG3 lncRNAs and lower levels of C3orf35, CASC2, and MEG3, and connected with ionization radiation response.

**lncRNA**	**Enrichemnt in lncRNA-expressing group**	**Gene**	**Process/Pathway**
**C3ORF35**	**Low**	**IQGAP2**	**UV_RESPONSE_DN**
**C3ORF35**	**Low**	**ELK4**	**UV_RESPONSE_DN**
**C3ORF35**	**Low**	**INPP4B**	**UV_RESPONSE_DN**
**C3ORF35**	**Low**	**AKAP12**	**UV_RESPONSE_DN**

C3ORF35	Low	ABHD2	UV_RESPONSE_DN
C3ORF35	Low	STEAP4	UV_RESPONSE_DN
C3ORF35	Low	MERTK	UV_RESPONSE_DN
C3ORF35	Low	AZGP1	UV_RESPONSE_DN
C3ORF35	Low	HERC3	UV_RESPONSE_DN
C3ORF35	Low	KLK3	UV_RESPONSE_DN
C3ORF35	Low	ZMIZ1	UV_RESPONSE_DN
C3ORF35	Low	ARID5B	UV_RESPONSE_DN
C3ORF35	Low	CDK6	UV_RESPONSE_DN
C3ORF35	Low	SLC26A2	UV_RESPONSE_DN
C3ORF35	Low	ABCC4	UV_RESPONSE_DN
C3ORF35	Low	ADAMTS1	UV_RESPONSE_DN
C3ORF35	Low	HPGD	UV_RESPONSE_DN
C3ORF35	Low	RPS6KA3	UV_RESPONSE_DN
C3ORF35	Low	NCOA4	UV_RESPONSE_DN
C3ORF35	Low	PDLIM5	UV_RESPONSE_DN
C3ORF35	Low	PTPN21	UV_RESPONSE_DN
C3ORF35	Low	SORD	UV_RESPONSE_DN
C3ORF35	Low	MAP7	UV_RESPONSE_DN
C3ORF35	Low	ELL2	UV_RESPONSE_DN
C3ORF35	Low	PIAS1	UV_RESPONSE_DN
C3ORF35	Low	SEC24D	UV_RESPONSE_DN
C3ORF35	Low	ALDH1A3	UV_RESPONSE_DN
C3ORF35	Low	APPBP2	UV_RESPONSE_DN
C3ORF35	Low	PGM3	UV_RESPONSE_DN
C3ORF35	Low	ITGAV	UV_RESPONSE_DN
C3ORF35	Low	HMGCR	UV_RESPONSE_DN
C3ORF35	Low	MAF	UV_RESPONSE_DN
C3ORF35	Low	KRT19	UV_RESPONSE_DN
C3ORF35	Low	GSR	UV_RESPONSE_DN
C3ORF35	Low	LMAN1	UV_RESPONSE_DN
C3ORF35	Low	ACTN1	UV_RESPONSE_DN
C3ORF35	Low	B4GALT1	UV_RESPONSE_DN
C3ORF35	Low	CDC14B	UV_RESPONSE_DN
C3ORF35	Low	SLC38A2	UV_RESPONSE_DN
C3ORF35	Low	SCD	UV_RESPONSE_DN
C3ORF35	Low	NDRG1	UV_RESPONSE_DN
C3ORF35	Low	DHCR24	UV_RESPONSE_DN
C3ORF35	Low	ZBTB10	UV_RESPONSE_DN
C3ORF35	Low	UBE2J1	UV_RESPONSE_DN
C3ORF35	Low	ELOVL5	UV_RESPONSE_DN
C3ORF35	Low	RAPGEF6	MITOTIC_SPINDLE
C3ORF35	Low	LATS1	MITOTIC_SPINDLE
C3ORF35	Low	RASA2	MITOTIC_SPINDLE

C3ORF35	Low	DOCK2	MITOTIC_SPINDLE
C3ORF35	Low	APC	MITOTIC_SPINDLE
C3ORF35	Low	BRCA2	MITOTIC_SPINDLE
C3ORF35	Low	DOCK4	MITOTIC_SPINDLE
C3ORF35	Low	OPHN1	MITOTIC_SPINDLE
C3ORF35	Low	NOTCH2	MITOTIC_SPINDLE
C3ORF35	Low	ARHGEF12	MITOTIC_SPINDLE
C3ORF35	Low	PREX1	MITOTIC_SPINDLE
C3ORF35	Low	SORBS2	MITOTIC_SPINDLE
C3ORF35	Low	PPP4R2	MITOTIC_SPINDLE
C3ORF35	Low	FGD6	MITOTIC_SPINDLE
C3ORF35	Low	ARHGAP29	MITOTIC_SPINDLE
C3ORF35	Low	INCENP	MITOTIC_SPINDLE
C3ORF35	Low	TRIO	MITOTIC_SPINDLE
C3ORF35	Low	CCDC88A	MITOTIC_SPINDLE
C3ORF35	Low	NF1	MITOTIC_SPINDLE
C3ORF35	Low	KIF20B	MITOTIC_SPINDLE
C3ORF35	Low	ALMS1	MITOTIC_SPINDLE
C3ORF35	Low	SSH2	MITOTIC_SPINDLE
C3ORF35	Low	ROCK1	MITOTIC_SPINDLE
C3ORF35	Low	ARHGAP5	MITOTIC_SPINDLE
C3ORF35	Low	KIF1B	MITOTIC_SPINDLE
C3ORF35	Low	SPTBN1	MITOTIC_SPINDLE
C3ORF35	Low	AKAP13	MITOTIC_SPINDLE
C3ORF35	Low	SMC1A	MITOTIC_SPINDLE
C3ORF35	Low	RALBP1	MITOTIC_SPINDLE
C3ORF35	Low	CLASP1	MITOTIC_SPINDLE
C3ORF35	Low	MYH10	MITOTIC_SPINDLE
C3ORF35	Low	RAPGEF5	MITOTIC_SPINDLE
C3ORF35	Low	SOS1	MITOTIC_SPINDLE
C3ORF35	Low	CLIP2	MITOTIC_SPINDLE
C3ORF35	Low	DST	MITOTIC_SPINDLE
C3ORF35	Low	WASF2	MITOTIC_SPINDLE
C3ORF35	Low	DYNC1H1	MITOTIC_SPINDLE
C3ORF35	Low	SHROOM2	MITOTIC_SPINDLE
C3ORF35	Low	PDLIM5	MITOTIC_SPINDLE
C3ORF35	Low	VCL	MITOTIC_SPINDLE
C3ORF35	Low	MYH9	MITOTIC_SPINDLE
C3ORF35	Low	TIAM1	MITOTIC_SPINDLE
C3ORF35	Low	CKAP5	MITOTIC_SPINDLE
C3ORF35	Low	SMC3	MITOTIC_SPINDLE
C3ORF35	Low	HOOK3	MITOTIC_SPINDLE
C3ORF35	Low	FGD4	MITOTIC_SPINDLE
C3ORF35	Low	MYO1E	MITOTIC_SPINDLE

C3ORF35	Low	SPTAN1	MITOTIC_SPINDLE
C3ORF35	Low	CLIP1	MITOTIC_SPINDLE
C3ORF35	Low	NIN	MITOTIC_SPINDLE
C3ORF35	Low	ECT2	MITOTIC_SPINDLE
C3ORF35	Low	EPB41L2	MITOTIC_SPINDLE
C3ORF35	Low	MID1	MITOTIC_SPINDLE
C3ORF35	Low	CDC42BPA	MITOTIC_SPINDLE
C3ORF35	Low	RASA1	MITOTIC_SPINDLE
C3ORF35	Low	FLNA	MITOTIC_SPINDLE
C3ORF35	Low	WASL	MITOTIC_SPINDLE
C3ORF35	Low	RAB3GAP1	MITOTIC_SPINDLE
C3ORF35	Low	ARFGEF1	MITOTIC_SPINDLE
C3ORF35	Low	ARHGEF11	MITOTIC_SPINDLE
C3ORF35	Low	CEP192	MITOTIC_SPINDLE
C3ORF35	Low	EPB41	MITOTIC_SPINDLE
C3ORF35	Low	RICTOR	MITOTIC_SPINDLE
C3ORF35	Low	KIF5B	MITOTIC_SPINDLE
C3ORF35	Low	NUMA1	MITOTIC_SPINDLE
C3ORF35	Low	LMNB1	MITOTIC_SPINDLE
C3ORF35	Low	SYNPO	MITOTIC_SPINDLE
C3ORF35	Low	DYNLL2	MITOTIC_SPINDLE
C3ORF35	Low	ABL1	MITOTIC_SPINDLE
C3ORF35	Low	CENPF	MITOTIC_SPINDLE
C3ORF35	Low	RASAL2	MITOTIC_SPINDLE
C3ORF35	Low	KIF3B	MITOTIC_SPINDLE
C3ORF35	Low	ANLN	MITOTIC_SPINDLE
C3ORF35	Low	KIF3C	MITOTIC_SPINDLE
C3ORF35	Low	TAOK2	MITOTIC_SPINDLE
C3ORF35	Low	CDC27	MITOTIC_SPINDLE
C3ORF35	Low	CD2AP	MITOTIC_SPINDLE
C3ORF35	Low	ACTN4	MITOTIC_SPINDLE
C3ORF35	Low	RFC1	MITOTIC_SPINDLE
C3ORF35	Low	FLNB	MITOTIC_SPINDLE
C3ORF35	Low	ITSN1	MITOTIC_SPINDLE
C3ORF35	Low	ABR	MITOTIC_SPINDLE
C3ORF35	Low	PLEKHG2	MITOTIC_SPINDLE
C3ORF35	Low	TOP2A	MITOTIC_SPINDLE
C3ORF35	Low	DLG1	MITOTIC_SPINDLE
C3ORF35	Low	KIF11	MITOTIC_SPINDLE
C3ORF35	Low	TUBGCP3	MITOTIC_SPINDLE
C3ORF35	Low	MYO9B	MITOTIC_SPINDLE
C3ORF35	Low	BCL2L11	MITOTIC_SPINDLE
C3ORF35	Low	ALS2	MITOTIC_SPINDLE
C3ORF35	Low	PCM1	MITOTIC_SPINDLE

C3ORF35	Low	SMC4	MITOTIC_SPINDLE
C3ORF35	Low	NCK2	MITOTIC_SPINDLE
C3ORF35	Low	PKD2	MITOTIC_SPINDLE
C3ORF35	Low	TUBA4A	MITOTIC_SPINDLE
C3ORF35	Low	RABGAP1	MITOTIC_SPINDLE
C3ORF35	Low	PCGF5	MITOTIC_SPINDLE
C3ORF35	Low	CENPE	MITOTIC_SPINDLE
C3ORF35	Low	PALLD	MITOTIC_SPINDLE
C3ORF35	Low	WASF1	MITOTIC_SPINDLE
C3ORF35	Low	PAFAH1B1	MITOTIC_SPINDLE
C3ORF35	Low	LLGL1	MITOTIC_SPINDLE
CASC2	Low	KLK8	P53_PATHWAY
CASC2	Low	CLCA2	P53_PATHWAY
CASC2	Low	SLC7A11	P53_PATHWAY
CASC2	Low	UPP1	P53_PATHWAY
CASC2	Low	PLK2	P53_PATHWAY
CASC2	Low	KRT17	P53_PATHWAY
CASC2	Low	BMP2	P53_PATHWAY
CASC2	Low	NHLH2	P53_PATHWAY
CASC2	Low	ZNF365	P53_PATHWAY
CASC2	Low	INHBB	P53_PATHWAY
CASC2	Low	HBEGF	P53_PATHWAY
CASC2	Low	TGFA	P53_PATHWAY
CASC2	Low	TNFSF9	P53_PATHWAY
CASC2	Low	MXD1	P53_PATHWAY
CASC2	Low	OSGIN1	P53_PATHWAY
CASC2	Low	ADA	P53_PATHWAY
CASC2	Low	SFN	P53_PATHWAY
CASC2	Low	IL1A	P53_PATHWAY
CASC2	Low	SERPINB5	P53_PATHWAY
CASC2	Low	NDRG1	P53_PATHWAY
CASC2	Low	CASP1	P53_PATHWAY
CASC2	Low	BAK1	P53_PATHWAY
CASC2	Low	RRAD	P53_PATHWAY
CASC2	Low	TGFB1	P53_PATHWAY
CASC2	Low	PROCR	P53_PATHWAY
CASC2	Low	TNNI1	P53_PATHWAY
CASC2	Low	CDH13	P53_PATHWAY
CASC2	Low	RAP2B	P53_PATHWAY
CASC2	Low	TRIB3	P53_PATHWAY
CASC2	Low	ST14	P53_PATHWAY
CASC2	Low	SLC19A2	P53_PATHWAY
CASC2	Low	PVT1	P53_PATHWAY
CASC2	Low	AK1	P53_PATHWAY

CASC2	Low	CDKN1A	P53_PATHWAY
CASC2	Low	CDK5R1	P53_PATHWAY
CASC2	Low	FOS	P53_PATHWAY
CASC2	Low	VDR	P53_PATHWAY
CASC2	Low	GM2A	P53_PATHWAY
CASC2	Low	KIF13B	P53_PATHWAY
CASC2	Low	S100A10	P53_PATHWAY
CASC2	Low	PLK3	P53_PATHWAY
CASC2	Low	HMOX1	P53_PATHWAY
CASC2	Low	SLC3A2	P53_PATHWAY
CASC2	Low	TM4SF1	P53_PATHWAY
CASC2	Low	CD82	P53_PATHWAY
CASC2	Low	PERP	P53_PATHWAY
CASC2	Low	ITGB4	P53_PATHWAY
CASC2	Low	SDC1	P53_PATHWAY
CASC2	Low	JUN	P53_PATHWAY
CASC2	Low	TAX1BP3	P53_PATHWAY
CASC2	Low	CEBPA	P53_PATHWAY
CASC2	Low	CCNK	P53_PATHWAY
CASC2	Low	CDKN2B	P53_PATHWAY
CASC2	Low	HRAS	P53_PATHWAY
CASC2	Low	NUDT15	P53_PATHWAY
CASC2	Low	EPS8L2	P53_PATHWAY
CASC2	Low	BAIAP2	P53_PATHWAY
CASC2	Low	AEN	P53_PATHWAY
CASC2	Low	EPHA2	P53_PATHWAY
CASC2	Low	RHBDF2	P53_PATHWAY
CASC2	Low	IER3	P53_PATHWAY
CASC2	Low	PTPRE	P53_PATHWAY
CASC2	Low	EI24	P53_PATHWAY
CASC2	Low	TAP1	P53_PATHWAY
CASC2	Low	PPP1R15A	P53_PATHWAY
CASC2	Low	FDXR	P53_PATHWAY
CASC2	Low	JAG2	P53_PATHWAY
CASC2	Low	ALOX15B	P53_PATHWAY
CASC2	Low	MAPKAPK3	P53_PATHWAY
CASC2	Low	PHLDA3	P53_PATHWAY
CASC2	Low	GADD45A	P53_PATHWAY
CASC2	Low	PDGFA	P53_PATHWAY
CASC2	Low	IER5	P53_PATHWAY
CASC2	Low	MXD4	P53_PATHWAY
CASC2	Low	APP	P53_PATHWAY
CASC2	Low	DDIT4	P53_PATHWAY
CASC2	Low	CD81	P53_PATHWAY

CASC2	Low	PITPNC1	P53_PATHWAY
CASC2	Low	CTSD	P53_PATHWAY
CASC2	Low	F2R	P53_PATHWAY
CASC2	Low	VWA5A	P53_PATHWAY
CASC2	Low	CCND2	P53_PATHWAY
CASC2	Low	TCHH	P53_PATHWAY
CASC2	Low	ZFP36L1	P53_PATHWAY
CASC2	Low	LIF	P53_PATHWAY
MEG3	Low	RAPGEF6	MITOTIC_SPINDLE
MEG3	Low	BRCA2	MITOTIC_SPINDLE
MEG3	Low	RASA2	MITOTIC_SPINDLE
MEG3	Low	LATS1	MITOTIC_SPINDLE
MEG3	Low	PPP4R2	MITOTIC_SPINDLE
MEG3	Low	INCENP	MITOTIC_SPINDLE
MEG3	Low	OPHN1	MITOTIC_SPINDLE
MEG3	Low	KIF20B	MITOTIC_SPINDLE
MEG3	Low	ESPL1	MITOTIC_SPINDLE
MEG3	Low	LMNB1	MITOTIC_SPINDLE
MEG3	Low	SASS6	MITOTIC_SPINDLE
MEG3	Low	FGD6	MITOTIC_SPINDLE
MEG3	Low	SHROOM2	MITOTIC_SPINDLE
MEG3	Low	ARHGAP5	MITOTIC_SPINDLE
MEG3	Low	EPB41	MITOTIC_SPINDLE
MEG3	Low	KIF11	MITOTIC_SPINDLE
MEG3	Low	NOTCH2	MITOTIC_SPINDLE
MEG3	Low	TIAM1	MITOTIC_SPINDLE
MEG3	Low	SMC3	MITOTIC_SPINDLE
MEG3	Low	WASF2	MITOTIC_SPINDLE
MEG3	Low	DOCK2	MITOTIC_SPINDLE
MEG3	Low	ARHGEF3	MITOTIC_SPINDLE
MEG3	Low	TTK	MITOTIC_SPINDLE
MEG3	Low	APC	MITOTIC_SPINDLE
MEG3	Low	ARHGEF12	MITOTIC_SPINDLE
MEG3	Low	SOS1	MITOTIC_SPINDLE
MEG3	Low	ROCK1	MITOTIC_SPINDLE
MEG3	Low	BUB1	MITOTIC_SPINDLE
MEG3	Low	NUSAP1	MITOTIC_SPINDLE
MEG3	Low	ANLN	MITOTIC_SPINDLE
MEG3	Low	KIF15	MITOTIC_SPINDLE
MEG3	Low	FGD4	MITOTIC_SPINDLE
MEG3	Low	RALBP1	MITOTIC_SPINDLE
MEG3	Low	PCGF5	MITOTIC_SPINDLE
MEG3	Low	ECT2	MITOTIC_SPINDLE
MEG3	Low	CENPF	MITOTIC_SPINDLE

MEG3	Low	TOP2A	MITOTIC_SPINDLE
MEG3	Low	BCL2L11	MITOTIC_SPINDLE
MEG3	Low	CDC42EP4	MITOTIC_SPINDLE
MEG3	Low	CEP192	MITOTIC_SPINDLE
MEG3	Low	RHOF	MITOTIC_SPINDLE
MEG3	Low	ARFGEF1	MITOTIC_SPINDLE
MEG3	Low	FBXO5	MITOTIC_SPINDLE
MEG3	Low	TUBGCP3	MITOTIC_SPINDLE
MEG3	Low	RAB3GAP1	MITOTIC_SPINDLE
MEG3	Low	RFC1	MITOTIC_SPINDLE
MEG3	Low	WASF1	MITOTIC_SPINDLE
MEG3	Low	ARF6	MITOTIC_SPINDLE
MEG3	Low	CKAP5	MITOTIC_SPINDLE
MEG3	Low	NF1	MITOTIC_SPINDLE
MEG3	Low	SMC4	MITOTIC_SPINDLE
MEG3	Low	PRC1	MITOTIC_SPINDLE
MEG3	Low	CENPE	MITOTIC_SPINDLE
MEG3	Low	SMC1A	MITOTIC_SPINDLE
MEG3	Low	FLNB	MITOTIC_SPINDLE
MEG3	Low	TPX2	MITOTIC_SPINDLE
MEG3	Low	LLGL1	MITOTIC_SPINDLE
MEG3	Low	NET1	MITOTIC_SPINDLE
MEG3	Low	KIF5B	MITOTIC_SPINDLE
MEG3	Low	CLIP1	MITOTIC_SPINDLE
MEG3	Low	KIF4A	MITOTIC_SPINDLE
MEG3	Low	ARHGAP27	MITOTIC_SPINDLE
MEG3	Low	CD2AP	MITOTIC_SPINDLE
MEG3	Low	RACGAP1	MITOTIC_SPINDLE
MEG3	Low	ALMS1	MITOTIC_SPINDLE
MEG3	Low	NUMA1	MITOTIC_SPINDLE
MEG3	Low	CDK1	MITOTIC_SPINDLE
MEG3	Low	SPTBN1	MITOTIC_SPINDLE
MEG3	Low	RAPGEF5	MITOTIC_SPINDLE
MEG3	Low	RASA1	MITOTIC_SPINDLE
MEG3	Low	KIF23	MITOTIC_SPINDLE
MEG3	Low	WASL	MITOTIC_SPINDLE
MEG3	Low	KIF2C	MITOTIC_SPINDLE
MEG3	Low	ABI1	MITOTIC_SPINDLE
MEG3	Low	NDC80	MITOTIC_SPINDLE
MEG3	Low	PDLIM5	MITOTIC_SPINDLE
MEG3	Low	AURKA	MITOTIC_SPINDLE
MEG3	Low	KIF3B	MITOTIC_SPINDLE
MEG3	Low	MYO1E	MITOTIC_SPINDLE
MEG3	Low	CCDC88A	MITOTIC_SPINDLE

MEG3	Low	SORBS2	MITOTIC_SPINDLE
MEG3	Low	TLK1	MITOTIC_SPINDLE
MEG3	Low	HOOK3	MITOTIC_SPINDLE
MEG3	Low	DLGAP5	MITOTIC_SPINDLE
MEG3	Low	MAPRE1	MITOTIC_SPINDLE
MEG3	Low	PREX1	MITOTIC_SPINDLE
MEG3	Low	VCL	MITOTIC_SPINDLE
MEG3	Low	TRIO	MITOTIC_SPINDLE
MEG3	Low	RICTOR	MITOTIC_SPINDLE
MEG3	Low	ACTN4	MITOTIC_SPINDLE
MEG3	Low	SPTAN1	MITOTIC_SPINDLE
MEG3	Low	ARHGEF11	MITOTIC_SPINDLE
MEG3	Low	ITSN1	MITOTIC_SPINDLE
MEG3	Low	KIF1B	MITOTIC_SPINDLE
MEG3	Low	RANBP9	MITOTIC_SPINDLE
MEG3	Low	NCK1	MITOTIC_SPINDLE
MEG3	Low	CEP250	MITOTIC_SPINDLE
MEG3	Low	NEK2	MITOTIC_SPINDLE
MEG3	Low	CENPJ	MITOTIC_SPINDLE
MEG3	Low	STAU1	MITOTIC_SPINDLE
MEG3	Low	DST	MITOTIC_SPINDLE
MEG3	Low	ABR	MITOTIC_SPINDLE
MEG3	Low	NCK2	MITOTIC_SPINDLE
MEG3	Low	DLG1	MITOTIC_SPINDLE
MEG3	Low	SSH2	MITOTIC_SPINDLE
MEG3	Low	CDC42BPA	MITOTIC_SPINDLE
MEG3	Low	KNTC1	MITOTIC_SPINDLE
MEG3	Low	CLASP1	MITOTIC_SPINDLE
MEG3	Low	EZR	MITOTIC_SPINDLE
MEG3	Low	RABGAP1	MITOTIC_SPINDLE
MEG3	Low	NIN	MITOTIC_SPINDLE
MEG3	Low	LRPPRC	MITOTIC_SPINDLE
MEG3	Low	TAOK2	MITOTIC_SPINDLE
MEG3	Low	PLK1	MITOTIC_SPINDLE
MEG3	Low	DYNLL2	MITOTIC_SPINDLE
MEG3	Low	CCNB2	MITOTIC_SPINDLE
MEG3	Low	PAFAH1B1	MITOTIC_SPINDLE
MEG3	Low	CDC27	MITOTIC_SPINDLE
MEG3	Low	PCM1	MITOTIC_SPINDLE
MEG3	Low	GEMIN4	MITOTIC_SPINDLE
MEG3	Low	CEP72	MITOTIC_SPINDLE
MEG3	Low	BARD1	G2M_CHECKPOINT
MEG3	Low	CCNT1	G2M_CHECKPOINT
MEG3	Low	BRCA2	G2M_CHECKPOINT

MEG3	Low	STIL	G2M_CHECKPOINT
MEG3	Low	RBL1	G2M_CHECKPOINT
MEG3	Low	SLC38A1	G2M_CHECKPOINT
MEG3	Low	RPS6KA5	G2M_CHECKPOINT
MEG3	Low	PTTG3P	G2M_CHECKPOINT
MEG3	Low	INCENP	G2M_CHECKPOINT
MEG3	Low	CDKN2C	G2M_CHECKPOINT
MEG3	Low	MKI67	G2M_CHECKPOINT
MEG3	Low	CASP8AP2	G2M_CHECKPOINT
MEG3	Low	KIF20B	G2M_CHECKPOINT
MEG3	Low	ESPL1	G2M_CHECKPOINT
MEG3	Low	CDC25A	G2M_CHECKPOINT
MEG3	Low	LMNB1	G2M_CHECKPOINT
MEG3	Low	KIF11	G2M_CHECKPOINT
MEG3	Low	TMPO	G2M_CHECKPOINT
MEG3	Low	NOTCH2	G2M_CHECKPOINT
MEG3	Low	PURA	G2M_CHECKPOINT
MEG3	Low	SLC7A1	G2M_CHECKPOINT
MEG3	Low	TTK	G2M_CHECKPOINT
MEG3	Low	CDC7	G2M_CHECKPOINT
MEG3	Low	HMMR	G2M_CHECKPOINT
MEG3	Low	BUB1	G2M_CHECKPOINT
MEG3	Low	NUSAP1	G2M_CHECKPOINT
MEG3	Low	STAG1	G2M_CHECKPOINT
MEG3	Low	CHEK1	G2M_CHECKPOINT
MEG3	Low	CCNA2	G2M_CHECKPOINT
MEG3	Low	SLC7A5	G2M_CHECKPOINT
MEG3	Low	KIF15	G2M_CHECKPOINT
MEG3	Low	CENPF	G2M_CHECKPOINT
MEG3	Low	EXO1	G2M_CHECKPOINT
MEG3	Low	CCNF	G2M_CHECKPOINT
MEG3	Low	TOP2A	G2M_CHECKPOINT
MEG3	Low	PML	G2M_CHECKPOINT
MEG3	Low	SMARCC1	G2M_CHECKPOINT
MEG3	Low	TOP1	G2M_CHECKPOINT
MEG3	Low	PLK4	G2M_CHECKPOINT
MEG3	Low	SMC2	G2M_CHECKPOINT
MEG3	Low	FBXO5	G2M_CHECKPOINT
MEG3	Low	DBF4	G2M_CHECKPOINT
MEG3	Low	SMC4	G2M_CHECKPOINT
MEG3	Low	PRC1	G2M_CHECKPOINT
MEG3	Low	ATRX	G2M_CHECKPOINT
MEG3	Low	CENPE	G2M_CHECKPOINT
MEG3	Low	SMC1A	G2M_CHECKPOINT

MEG3	Low	EZH2	G2M_CHECKPOINT
MEG3	Low	TPX2	G2M_CHECKPOINT
MEG3	Low	ATF5	G2M_CHECKPOINT
MEG3	Low	KIF5B	G2M_CHECKPOINT
MEG3	Low	KIF4A	G2M_CHECKPOINT
MEG3	Low	POLQ	G2M_CHECKPOINT
MEG3	Low	G3BP1	G2M_CHECKPOINT
MEG3	Low	NUP98	G2M_CHECKPOINT
MEG3	Low	RACGAP1	G2M_CHECKPOINT
MEG3	Low	HIF1A	G2M_CHECKPOINT
MEG3	Low	DR1	G2M_CHECKPOINT
MEG3	Low	SYNCRIP	G2M_CHECKPOINT
MEG3	Low	NUMA1	G2M_CHECKPOINT
MEG3	Low	CDK1	G2M_CHECKPOINT
MEG3	Low	AMD1	G2M_CHECKPOINT
MEG3	Low	KIF23	G2M_CHECKPOINT
MEG3	Low	TFDP1	G2M_CHECKPOINT
MEG3	Low	POLE	G2M_CHECKPOINT
MEG3	Low	MYBL2	G2M_CHECKPOINT
MEG3	Low	KIF2C	G2M_CHECKPOINT
MEG3	Low	NDC80	G2M_CHECKPOINT
MEG3	Low	AURKA	G2M_CHECKPOINT
MEG3	Low	PBK	G2M_CHECKPOINT
MEG3	Low	CTCF	G2M_CHECKPOINT
MEG3	Low	BCL3	G2M_CHECKPOINT
MEG3	Low	RAD21	G2M_CHECKPOINT
MEG3	Low	WRN	G2M_CHECKPOINT
MEG3	Low	ARID4A	G2M_CHECKPOINT
MEG3	Low	MTF2	G2M_CHECKPOINT
MEG3	Low	MCM2	G2M_CHECKPOINT
MEG3	Low	HOXC10	G2M_CHECKPOINT
MEG3	Low	KPNB1	G2M_CHECKPOINT
MEG3	Low	E2F2	G2M_CHECKPOINT
MEG3	Low	CHAF1A	G2M_CHECKPOINT
MEG3	Low	PDS5B	G2M_CHECKPOINT
MEG3	Low	NOLC1	G2M_CHECKPOINT
MEG3	Low	HNRNPU	G2M_CHECKPOINT
MEG3	Low	NEK2	G2M_CHECKPOINT
MEG3	Low	RBM14	G2M_CHECKPOINT
MEG3	Low	SMAD3	G2M_CHECKPOINT
MEG3	Low	CDC25B	G2M_CHECKPOINT
MEG3	Low	MCM6	G2M_CHECKPOINT
MEG3	Low	TLE3	G2M_CHECKPOINT
MEG3	Low	CDC20	G2M_CHECKPOINT

MEG3	Low	GSPT1	G2M_CHECKPOINT
MEG3	Low	MYC	G2M_CHECKPOINT
MEG3	Low	ODC1	G2M_CHECKPOINT
MEG3	Low	BUB3	G2M_CHECKPOINT
MEG3	Low	LBR	G2M_CHECKPOINT
MEG3	Low	SQLE	G2M_CHECKPOINT
MEG3	Low	SFPQ	G2M_CHECKPOINT
MEG3	Low	KPNA2	G2M_CHECKPOINT
MEG3	Low	TRA2B	G2M_CHECKPOINT
MEG3	Low	PLK1	G2M_CHECKPOINT
MEG3	Low	HIRA	G2M_CHECKPOINT
MEG3	Low	UPF1	G2M_CHECKPOINT
MEG3	Low	CCNB2	G2M_CHECKPOINT
MEG3	Low	PAFAH1B1	G2M_CHECKPOINT
MEG3	Low	CDC27	G2M_CHECKPOINT
SFTA1P	High	THBD	ANGIOGENESIS
SFTA1P	High	CCND2	ANGIOGENESIS
SFTA1P	High	SPP1	ANGIOGENESIS
SFTA1P	High	NRP1	ANGIOGENESIS
SFTA1P	High	PF4	ANGIOGENESIS
SFTA1P	High	LRPAP1	ANGIOGENESIS
SFTA1P	High	JAG1	ANGIOGENESIS
SFTA1P	High	TNFRSF21	ANGIOGENESIS
SFTA1P	High	FGFR1	ANGIOGENESIS
SFTA1P	High	APP	ANGIOGENESIS
SFTA1P	High	JAG2	ANGIOGENESIS
SFTA1P	High	PDGFA	ANGIOGENESIS
SFTA1P	High	PRG2	ANGIOGENESIS
SFTA1P	High	PGLYRP1	ANGIOGENESIS
SFTA1P	High	TIMP1	ANGIOGENESIS
SFTA1P	High	LUM	ANGIOGENESIS
SFTA1P	High	VAV2	ANGIOGENESIS
SFTA1P	High	KCNJ8	ANGIOGENESIS
SFTA1P	High	COL3A1	ANGIOGENESIS
SFTA1P	High	VCAN	ANGIOGENESIS
SFTA1P	High	FSTL1	ANGIOGENESIS
SFTA1P	High	POSTN	ANGIOGENESIS
SFTA1P	High	APOH	ANGIOGENESIS
SFTA1P	High	COL5A2	ANGIOGENESIS
SFTA1P	High	OLR1	ANGIOGENESIS
SFTA1P	High	VTN	ANGIOGENESIS
SFTA1P	High	MSX1	ANGIOGENESIS
SFTA1P	High	SGCG	EPITHELIAL_MESENCHYMAL_TRANSITION
SFTA1P	High	PLOD3	EPITHELIAL_MESENCHYMAL_TRANSITION

SFTA1P	High	LAMC1	EPITHELIAL_MESENCHYMAL_TRANSITION
SFTA1P	High	FMOD	EPITHELIAL_MESENCHYMAL_TRANSITION
SFTA1P	High	SDC4	EPITHELIAL_MESENCHYMAL_TRANSITION
SFTA1P	High	FLNA	EPITHELIAL_MESENCHYMAL_TRANSITION
SFTA1P	High	GADD45A	EPITHELIAL_MESENCHYMAL_TRANSITION
SFTA1P	High	VIM	EPITHELIAL_MESENCHYMAL_TRANSITION
SFTA1P	High	CALU	EPITHELIAL_MESENCHYMAL_TRANSITION
SFTA1P	High	COL4A1	EPITHELIAL_MESENCHYMAL_TRANSITION
SFTA1P	High	CD59	EPITHELIAL_MESENCHYMAL_TRANSITION
SFTA1P	High	COL4A2	EPITHELIAL_MESENCHYMAL_TRANSITION
SFTA1P	High	COL7A1	EPITHELIAL_MESENCHYMAL_TRANSITION
SFTA1P	High	PMP22	EPITHELIAL_MESENCHYMAL_TRANSITION
SFTA1P	High	IL32	EPITHELIAL_MESENCHYMAL_TRANSITION
SFTA1P	High	OXTR	EPITHELIAL_MESENCHYMAL_TRANSITION
SFTA1P	High	MMP14	EPITHELIAL_MESENCHYMAL_TRANSITION
SFTA1P	High	ITGB1	EPITHELIAL_MESENCHYMAL_TRANSITION
SFTA1P	High	GADD45B	EPITHELIAL_MESENCHYMAL_TRANSITION
SFTA1P	High	DCN	EPITHELIAL_MESENCHYMAL_TRANSITION
SFTA1P	High	FBN2	EPITHELIAL_MESENCHYMAL_TRANSITION
SFTA1P	High	TIMP1	EPITHELIAL_MESENCHYMAL_TRANSITION
SFTA1P	High	PRRX1	EPITHELIAL_MESENCHYMAL_TRANSITION
SFTA1P	High	RHOB	EPITHELIAL_MESENCHYMAL_TRANSITION
SFTA1P	High	LUM	EPITHELIAL_MESENCHYMAL_TRANSITION
SFTA1P	High	PLOD1	EPITHELIAL_MESENCHYMAL_TRANSITION
SFTA1P	High	TGFB1	EPITHELIAL_MESENCHYMAL_TRANSITION
SFTA1P	High	THY1	EPITHELIAL_MESENCHYMAL_TRANSITION
SFTA1P	High	PVR	EPITHELIAL_MESENCHYMAL_TRANSITION
SFTA1P	High	CDH11	EPITHELIAL_MESENCHYMAL_TRANSITION
SFTA1P	High	COL6A3	EPITHELIAL_MESENCHYMAL_TRANSITION
SFTA1P	High	PDGFRB	EPITHELIAL_MESENCHYMAL_TRANSITION
SFTA1P	High	IL6	EPITHELIAL_MESENCHYMAL_TRANSITION
SFTA1P	High	GJA1	EPITHELIAL_MESENCHYMAL_TRANSITION
SFTA1P	High	ANPEP	EPITHELIAL_MESENCHYMAL_TRANSITION
SFTA1P	High	FBN1	EPITHELIAL_MESENCHYMAL_TRANSITION
SFTA1P	High	COL6A2	EPITHELIAL_MESENCHYMAL_TRANSITION
SFTA1P	High	COL3A1	EPITHELIAL_MESENCHYMAL_TRANSITION
SFTA1P	High	SPARC	EPITHELIAL_MESENCHYMAL_TRANSITION
SFTA1P	High	GREM1	EPITHELIAL_MESENCHYMAL_TRANSITION
SFTA1P	High	ACTA2	EPITHELIAL_MESENCHYMAL_TRANSITION
SFTA1P	High	VCAN	EPITHELIAL_MESENCHYMAL_TRANSITION
SFTA1P	High	ECM2	EPITHELIAL_MESENCHYMAL_TRANSITION
SFTA1P	High	COL1A2	EPITHELIAL_MESENCHYMAL_TRANSITION
SFTA1P	High	COL1A1	EPITHELIAL_MESENCHYMAL_TRANSITION
SFTA1P	High	ADAM12	EPITHELIAL_MESENCHYMAL_TRANSITION

SFTA1P	High	BMP1	EPITHELIAL_MESENCHYMAL_TRANSITION
SFTA1P	High	LRRC15	EPITHELIAL_MESENCHYMAL_TRANSITION
SFTA1P	High	FSTL1	EPITHELIAL_MESENCHYMAL_TRANSITION
SFTA1P	High	MXRA5	EPITHELIAL_MESENCHYMAL_TRANSITION
SFTA1P	High	SNAI2	EPITHELIAL_MESENCHYMAL_TRANSITION
SFTA1P	High	BGN	EPITHELIAL_MESENCHYMAL_TRANSITION
SFTA1P	High	NID2	EPITHELIAL_MESENCHYMAL_TRANSITION
SFTA1P	High	FERMT2	EPITHELIAL_MESENCHYMAL_TRANSITION
SFTA1P	High	CALD1	EPITHELIAL_MESENCHYMAL_TRANSITION
SFTA1P	High	EFEMP2	EPITHELIAL_MESENCHYMAL_TRANSITION
SFTA1P	High	POSTN	EPITHELIAL_MESENCHYMAL_TRANSITION
SFTA1P	High	TPM2	EPITHELIAL_MESENCHYMAL_TRANSITION
SFTA1P	High	LAMA1	EPITHELIAL_MESENCHYMAL_TRANSITION
SFTA1P	High	DKK1	EPITHELIAL_MESENCHYMAL_TRANSITION
SFTA1P	High	TNC	EPITHELIAL_MESENCHYMAL_TRANSITION
SFTA1P	High	FOXC2	EPITHELIAL_MESENCHYMAL_TRANSITION
SFTA1P	High	LGALS1	EPITHELIAL_MESENCHYMAL_TRANSITION
SFTA1P	High	TFPI2	EPITHELIAL_MESENCHYMAL_TRANSITION
SFTA1P	High	MMP2	EPITHELIAL_MESENCHYMAL_TRANSITION
SFTA1P	High	HTRA1	EPITHELIAL_MESENCHYMAL_TRANSITION
SFTA1P	High	MYL9	EPITHELIAL_MESENCHYMAL_TRANSITION
SFTA1P	High	PLAUR	EPITHELIAL_MESENCHYMAL_TRANSITION
SFTA1P	High	PCOLCE	EPITHELIAL_MESENCHYMAL_TRANSITION
SFTA1P	High	SCG2	EPITHELIAL_MESENCHYMAL_TRANSITION
SFTA1P	High	COL16A1	EPITHELIAL_MESENCHYMAL_TRANSITION
SFTA1P	High	ITGA5	EPITHELIAL_MESENCHYMAL_TRANSITION
SFTA1P	High	RGS4	EPITHELIAL_MESENCHYMAL_TRANSITION
SFTA1P	High	SERPINH1	EPITHELIAL_MESENCHYMAL_TRANSITION
SFTA1P	High	ITGB3	EPITHELIAL_MESENCHYMAL_TRANSITION
SFTA1P	High	LOXL2	EPITHELIAL_MESENCHYMAL_TRANSITION
SFTA1P	High	SGCD	EPITHELIAL_MESENCHYMAL_TRANSITION
SFTA1P	High	THBS2	EPITHELIAL_MESENCHYMAL_TRANSITION
SFTA1P	High	CAP2	EPITHELIAL_MESENCHYMAL_TRANSITION
SFTA1P	High	EMP3	EPITHELIAL_MESENCHYMAL_TRANSITION
SFTA1P	High	GLIPR1	EPITHELIAL_MESENCHYMAL_TRANSITION
SFTA1P	High	GAS1	EPITHELIAL_MESENCHYMAL_TRANSITION
SFTA1P	High	THBS1	EPITHELIAL_MESENCHYMAL_TRANSITION
SFTA1P	High	COL5A2	EPITHELIAL_MESENCHYMAL_TRANSITION
SFTA1P	High	TPM1	EPITHELIAL_MESENCHYMAL_TRANSITION
SFTA1P	High	COL5A3	EPITHELIAL_MESENCHYMAL_TRANSITION
SFTA1P	High	PMEPA1	EPITHELIAL_MESENCHYMAL_TRANSITION
SFTA1P	High	SERPINE2	EPITHELIAL_MESENCHYMAL_TRANSITION
SFTA1P	High	FN1	EPITHELIAL_MESENCHYMAL_TRANSITION
SFTA1P	High	LAMC2	EPITHELIAL_MESENCHYMAL_TRANSITION

SFTA1P	High	LAMA3	EPITHELIAL_MESENCHYMAL_TRANSITION
SFTA1P	High	COL5A1	EPITHELIAL_MESENCHYMAL_TRANSITION
SFTA1P	High	CTHRC1	EPITHELIAL_MESENCHYMAL_TRANSITION
SFTA1P	High	TAGLN	EPITHELIAL_MESENCHYMAL_TRANSITION
SFTA1P	High	COL11A1	EPITHELIAL_MESENCHYMAL_TRANSITION
SFTA1P	High	TNFRSF12A	EPITHELIAL_MESENCHYMAL_TRANSITION
SFTA1P	High	BASP1	EPITHELIAL_MESENCHYMAL_TRANSITION
SFTA1P	High	PTHLH	EPITHELIAL_MESENCHYMAL_TRANSITION
SFTA1P	High	COL12A1	EPITHELIAL_MESENCHYMAL_TRANSITION
SFTA1P	High	PTX3	EPITHELIAL_MESENCHYMAL_TRANSITION
SFTA1P	High	SERPINE1	EPITHELIAL_MESENCHYMAL_TRANSITION
SFTA1P	High	TIMP3	EPITHELIAL_MESENCHYMAL_TRANSITION
SFTA1P	High	NNMT	EPITHELIAL_MESENCHYMAL_TRANSITION
SFTA1P	High	NTM	EPITHELIAL_MESENCHYMAL_TRANSITION
SFTA1P	High	MSX1	EPITHELIAL_MESENCHYMAL_TRANSITION
SFTA1P	High	SPOCK1	EPITHELIAL_MESENCHYMAL_TRANSITION
SFTA1P	High	CDH2	EPITHELIAL_MESENCHYMAL_TRANSITION
SFTA1P	High	TGFBI	EPITHELIAL_MESENCHYMAL_TRANSITION
SFTA1P	High	AREG	EPITHELIAL_MESENCHYMAL_TRANSITION
SFTA1P	High	NT5E	EPITHELIAL_MESENCHYMAL_TRANSITION
SFTA1P	High	MMP3	EPITHELIAL_MESENCHYMAL_TRANSITION
SFTA1P	High	MATN3	EPITHELIAL_MESENCHYMAL_TRANSITION
SFTA1P	High	INHBA	EPITHELIAL_MESENCHYMAL_TRANSITION
SFTA1P	High	FSTL3	EPITHELIAL_MESENCHYMAL_TRANSITION
SFTA1P	High	MFAP5	EPITHELIAL_MESENCHYMAL_TRANSITION
SFTA1P	High	MMP1	EPITHELIAL_MESENCHYMAL_TRANSITION
SFTA1P	High	FAP	EPITHELIAL_MESENCHYMAL_TRANSITION
SFTA1P	High	VEGFC	EPITHELIAL_MESENCHYMAL_TRANSITION
SNHG3	High	DAD1	DNA_REPAIR
SNHG3	High	SNAPC5	DNA_REPAIR
SNHG3	High	ERCC1	DNA_REPAIR
SNHG3	High	GTF3C5	DNA_REPAIR
SNHG3	High	FEN1	DNA_REPAIR
SNHG3	High	RPA2	DNA_REPAIR
SNHG3	High	ADRM1	DNA_REPAIR
SNHG3	High	POLD1	DNA_REPAIR
SNHG3	High	SF3A3	DNA_REPAIR
SNHG3	High	RAD51	DNA_REPAIR
SNHG3	High	GTF2B	DNA_REPAIR
SNHG3	High	POLR2E	DNA_REPAIR
SNHG3	High	SUPT4H1	DNA_REPAIR
SNHG3	High	UPF3B	DNA_REPAIR
SNHG3	High	RAE1	DNA_REPAIR
SNHG3	High	CDA	DNA_REPAIR

SNHG3	High	HPRT1	DNA_REPAIR
SNHG3	High	RAD52	DNA_REPAIR
SNHG3	High	BRF2	DNA_REPAIR
SNHG3	High	RFC4	DNA_REPAIR
SNHG3	High	GUK1	DNA_REPAIR
SNHG3	High	CSTF3	DNA_REPAIR
SNHG3	High	TYMS	DNA_REPAIR
SNHG3	High	POLR1D	DNA_REPAIR
SNHG3	High	APRT	DNA_REPAIR
SNHG3	High	CETN2	DNA_REPAIR
SNHG3	High	POLR2J	DNA_REPAIR
SNHG3	High	GPX4	DNA_REPAIR
SNHG3	High	VPS28	DNA_REPAIR
SNHG3	High	AK1	DNA_REPAIR
SNHG3	High	MPG	DNA_REPAIR
SNHG3	High	TAF9	DNA_REPAIR
SNHG3	High	SURF1	DNA_REPAIR
SNHG3	High	TAF12	DNA_REPAIR
SNHG3	High	SNAPC4	DNA_REPAIR
SNHG3	High	EIF1B	DNA_REPAIR
SNHG3	High	POLL	DNA_REPAIR
SNHG3	High	ZNF707	DNA_REPAIR
SNHG3	High	GTF2A2	DNA_REPAIR
SNHG3	High	POLD4	DNA_REPAIR
SNHG3	High	POLR2K	DNA_REPAIR
SNHG3	High	IMPDH2	DNA_REPAIR
SNHG3	High	DUT	DNA_REPAIR
SNHG3	High	MRPL40	DNA_REPAIR
SNHG3	High	POLR2G	DNA_REPAIR
SNHG3	High	DGUOK	DNA_REPAIR
SNHG3	High	POLE4	DNA_REPAIR
SNHG3	High	EDF1	DNA_REPAIR
SNHG3	High	TAF10	DNA_REPAIR
SNHG3	High	TARBP2	DNA_REPAIR
SNHG3	High	ITPA	DNA_REPAIR
SNHG3	High	GTF2H5	DNA_REPAIR
SNHG3	High	TAF1C	DNA_REPAIR
SNHG3	High	POLB	DNA_REPAIR
SNHG3	High	NME4	DNA_REPAIR
SNHG3	High	NME1	DNA_REPAIR
SNHG3	High	POLR2I	DNA_REPAIR
SNHG3	High	RPA3	DNA_REPAIR
SNHG3	High	RBX1	DNA_REPAIR
SNHG3	High	POLR2H	DNA_REPAIR

**SNHG3**	**High**	**POLR2F**	**DNA_REPAIR**
**SNHG3**	**High**	**BOLA2**	**DNA_REPAIR**
**SNHG3**	**High**	**COX17**	**DNA_REPAIR**
**SNHG3**	**High**	**POLR1C**	**DNA_REPAIR**
**SNHG3**	**High**	**NT5C**	**DNA_REPAIR**
**SNHG3**	**High**	**SAC3D1**	**DNA_REPAIR**
**SNHG3**	**High**	**NME3**	**DNA_REPAIR**
**SNHG3**	**High**	**CCNO**	**DNA_REPAIR**

Table S8. Analysis of infiltration of immune cells into tumor tissues and infer tumor purity depending on the lncRNA expression level; $p < 0.05$ considered significant.

lncRNA	Parameter	Group	Minimum	Maximum	Range	Mean	Std. Deviation	Std. Error of Mean	p-value
**C3orf35**	Stromal_score	Low	-149266	146362	295628	-9884	55223	6554	0.3996
		High	-181593	164637	346230	-12486	45418	5280	
	Immune_score	Low	-118885	235128	354013	33297	65096	7672	0.1074
		High	-61871	205704	267575	18073	50863	5913	
	ESTIMATE_score	Low	-268151	263038	531189	21424	100892	11890	0.1218
		High	-235981	320647	556628	6241	70019	8140	
	Lymphocyte Infiltration Signature Score	Low	-1.657	2.387	4.044	0.31	0.9491	0.1119	0.3503
		High	-2.18	2.817	4.996	0.1512	1.091	0.1268	
**C5orf38**	Stromal_score	Low	-181593	164637	346230	-14186	60213	7146	0.989
		High	-135975	101927	237902	-8359	38690	4498	
	Immune_score	Low	-118885	235128	354013	41573	66943	7889	**<0.0001**
		High	-89345	157608	246953	10021	44362	5157	
	ESTIMATE_score	Low	-268151	320647	588798	28758	100802	11880	**0.0005**
		High	-179683	215983	395666	-894.9	67794	7881	
	Lymphocyte Infiltration Signature Score	Low	-1.138	2.817	3.955	0.6422	0.9318	0.1098	**<0.0001**
		High	-2.18	2.221	4.401	-0.172	0.9499	0.1104	
**MEG3**	Stromal_score	Low	-181593	146362	327955	-17341	56474	6655	**0.0122**
		High	-149266	164637	313903	-5167	42896	5021	
	Immune_score	Low	-61871	205704	267575	36568	62329	7346	0.1126

		High	-118885	235128	354013	14890	53017	6163	
	ESTIMATE_score	Low	-235981	263038	499019	10437	87816	10349	0.9735
		High	-268151	320647	588798	16931	86005	9998	
	Lymphocyte Infiltration Signature Score	Low	-1.659	2.817	4.476	0.4054	0.9955	0.1173	**0.0334**
		High	-2.18	2.301	4.48	0.05834	1.027	0.1194	
**MYCNOS**	Stromal_score	Low	-149266	146362	295628	-15205	49515	5876	0.8576
		High	-181593	164637	346230	-7381	51078	5938	
	Immune_score	Low	-118885	146468	265353	19475	49952	5887	0.4883
		High	-89345	235128	324473	31521	65764	7645	
	ESTIMATE_score	Low	-268151	215983	484134	2957	81443	9598	0.3496
		High	-235981	320647	556628	24209	90781	10553	
	Lymphocyte Infiltration Signature Score	Low	-1.832	2.301	4.133	0.04323	0.9109	0.1074	**0.0475**
		High	-2.18	2.817	4.996	0.4108	1.097	0.1275	
**SNHG3**	Stromal_score	Low	-141047	164637	305684	-872.9	52258	6202	0.078
		High	-181593	89465	271058	-21132	46562	5413	
	Immune_score	Low	-89345	205704	295049	35472	62161	7326	**0.0093**
		High	-118885	235128	354013	15957	53626	6234	
	ESTIMATE_score	Low	-164351	320647	484998	33408	93141	10977	**0.0013**
		High	-268151	262986	531137	-5419	75677	8797	
	Lymphocyte Infiltration Signature Score	Low	-2.18	2.817	4.996	0.4178	1.013	0.1193	**0.0243**
		High	-1.832	2.239	04.07	0.04632	1.006	0.1169	
**TMEM105**	Stromal_score	Low	-181593	164637	346230	-14914	56134	6615	0.5267
		High	-145278	146362	291640	-7561	43877	5135	
	Immune_score	Low	-118885	235128	354013	40799	71030	8371	**0.0268**
		High	-89345	157608	246953	10774	38297	4452	
	ESTIMATE_score	Low	-268151	320647	588798	26653	107677	12690	0.0714

		High	-164351	209395	373746	1154	57590	6695	
	Lymphocyte Infiltration Signature Score	Low	-1.659	2.817	4.476	0.5563	1.025	0.1208	**0.0002**
		High	-2.18	2.221	4.401	-0.08841	0.9208	0.107	
**CASC2**	Stromal_score	Low	-149266	99083	248349	-18354	51275	6085	0.1904
		High	-181593	164637	346230	-4360	48703	5662	
	Immune_score	Low	-118885	205704	324589	26677	62006	7308	0.5674
		High	-55276	235128	290404	24514	55520	6454	
	ESTIMATE_score	Low	-268151	263038	531189	9707	90680	10687	0.4201
		High	-235981	320647	556628	17641	83001	9649	
	Lymphocyte Infiltration Signature Score	Low	-1.659	2.817	4.476	0.3203	1.034	0.1219	0.3293
		High	-2.18	2.387	4.566	0.1411	1.011	0.1175	
**C10orf55**	Stromal_score	Low	-149266	164637	313903	-14198	53348	6287	0.8825
		High	-181593	146362	327955	-8267	47280	5534	
	Immune_score	Low	-118885	235128	354013	27905	60317	7108	0.5258
		High	-89345	205704	295049	23320	57230	6653	
	ESTIMATE_score	Low	-268151	320647	588798	20288	97106	11444	0.5834
		High	-235981	241058	477039	7347	75255	8748	
	Lymphocyte Infiltration Signature Score	Low	-1.732	2.387	4.119	0.2897	1.014	0.1195	0.5265
		High	-2.18	2.817	4.996	0.1709	1.035	0.1203	
**SFTA1P**	Stromal_score	Low	-181593	146362	327955	-15839	53263	6277	0.5139
		High	-145278	164637	309915	-6648	47112	5514	
	Immune_score	Low	-118885	235128	354013	32104	65709	7744	0.5942
		High	-89345	205704	295049	19234	50419	5861	
	ESTIMATE_score	Low	-268151	263038	531189	10372	93478	11017	0.7802
		High	-164351	320647	484998	16995	79985	9298	
		Low	-2.18	2.387	4.566	0.2283	1.039	0.1225	0.9728

	Lymphocyte Infiltration Signature Score	High	-1.832	2.817	4.648	0.2307	1.014	0.1178	
